# Personalized treatment options for chronic diseases using precision cohort analytics

**DOI:** 10.1038/s41598-021-80967-5

**Published:** 2021-01-13

**Authors:** Kenney Ng, Uri Kartoun, Harry Stavropoulos, John A. Zambrano, Paul C. Tang

**Affiliations:** 1 Center for Computational Health, IBM Research, 75 Binney Street, Cambridge, MA 02142 USA; 2 Atrius Health, Boston, MA USA; 3 Stanford Clinical Excellence Research Center, Stanford, CA USA

**Keywords:** Hypertension, Type 2 diabetes, Dyslipidaemias, Medical research, Health care, Data mining, Machine learning, Predictive medicine

## Abstract

To support point-of-care decision making by presenting outcomes of past treatment choices for cohorts of similar patients based on observational data from electronic health records (EHRs), a machine-learning precision cohort treatment option (PCTO) workflow consisting of (1) data extraction, (2) similarity model training, (3) precision cohort identification, and (4) treatment options analysis was developed. The similarity model is used to dynamically create a cohort of similar patients, to inform clinical decisions about an individual patient. The workflow was implemented using EHR data from a large health care provider for three different highly prevalent chronic diseases: hypertension (HTN), type 2 diabetes mellitus (T2DM), and hyperlipidemia (HL). A retrospective analysis demonstrated that treatment options with better outcomes were available for a majority of cases (75%, 74%, 85% for HTN, T2DM, HL, respectively). The models for HTN and T2DM were deployed in a pilot study with primary care physicians using it during clinic visits. A novel data-analytic workflow was developed to create patient-similarity models that dynamically generate personalized treatment insights at the point-of-care. By leveraging both knowledge-driven treatment guidelines and data-driven EHR data, physicians can incorporate real-world evidence in their medical decision-making process when considering treatment options for individual patients.

## Introduction

Management of chronic disease is a global challenge. In the US, 86% of people over 65 have at least one chronic condition^1^. To help manage the care of patients with chronic diseases, physicians use treatment guidelines, results of randomized clinical trials (RCTs), and their own patient-panel experience, to inform treatment decisions for individual patients. Treatment guidelines are usually based on the results of RCTs and expert opinions^2^. What is not widely appreciated, however, is that only a small minority of patients could actually meet the inclusion/exclusion eligibility criteria for clinical trials, thus substantially limiting the generalizability of the trial results and the applicability of common clinical guidelines^3,4^. Consequently, to make treatment decisions for individual patients, physicians often draw on their own experience, however the number of patients in their panel who are similar to a specific patient is very limited^5^. The rapid adoption of electronic health records (EHRs) has led to the capture of health information on millions of patients, with detailed longitudinal information including demographics, diagnoses, medications, diseases, laboratory observations, procedure orders, and associated outcomes^3,6,7^. Each medication order, laboratory order, or procedure order recorded in the EHR can be viewed as an intervention^8,9^. In this paper, we analyze the outcomes of individual patient medication treatment decisions.

Our approach provides information about the outcomes of similar patients in a similar clinical situation to the patient of interest, which we call a Precision Cohort, at the point-of-care. During a clinic visit, our system dynamically creates a precision cohort of clinically similar patient decision points. It then generates distributional and comparative statistics of the resulting outcomes of diverse treatment options administered to other patients in the precision cohort. We demonstrate the suitability of the approach by implementing it using EHR data from a large health care provider for three chronic diseases: hypertension (HTN), type 2 diabetes mellitus (T2DM), and hyperlipidemia (HL). A retrospective analysis demonstrated that treatment options with better outcomes were available for a majority of cases across the three diseases: 75%, 74%, 85% for HTN, T2DM, HL, respectively. The resultant models for HTN and T2DM were actively used by primary care physicians (PCPs) during clinic visits, in a pilot study.

## Background

Work on developing and applying patient similarity is not new^10,11^. Some work focused on the development of methods to compute patient similarity, to assist in identifying clinically similar patients: for example, by developing a locally supervised metric learning (LSML) method that can automatically learn disease-specific similarity metrics^12^, by applying multiple similarity criteria depending on variable types^13^, by analyzing images^14^, and by identifying genetic similarities^15^. Common approaches to identify groups of patients with similar time-dependent characteristics include time-series analysis methods^16–18^ and clustering techniques^19,20^. Other work has focused on the application of patient similarity to specific use cases: e.g., for predicting disease onset risk^21,22^, drug effectiveness^23^, identifying disease sub-phenotypes^24^, personalized risk factors^25^, personalizing blood glucose prediction^26^, and diagnosing chronic diseases^27^. There has also been work on clinical decision support (CDS) approaches that leverage patient similarity. These include:

- A system that uses clinical data (originating in notes and structured fields), to map a patient to a position in a clinical pathway (extracted from guidelines) to inform treatment^28^,
- A system for individualized cancer treatments, by predicting an individual’s responsiveness to different treatments, based on gene expression profiles^29^,
- A system to identify a patient as a candidate for a specific treatment, that includes methods to create patient clusters a priori, based on a collection of clinical characteristics with an associated desired treatment, as well as methods to efficiently create patient clusters ^30^, and
- A system that generates a patient trajectory graph from EHR data, capturing conditions, associated outcomes, and medical interventions, and suggests medical guidelines at patient group/cluster levels^31^.

Our precision cohort treatment options (PCTO) approach differs from these existing CDS approaches in the following ways:

1. It identifies and extracts appropriate disease-specific “clinical treatment decision points” from the longitudinal patient EHR data to use as the primary events of interest for modeling and analysis,
2. It selects variables for assessing patient similarity leveraging multiple sources of information: (a) variables associated with the propensity of receiving treatment derived from knowledge-driven clinical guidelines, and (b) variables associated with disease control outcomes using data-driven variable construction and feature selection from EHR data,
3. It dynamically creates, at runtime, a precision cohort of patient events that are clinically similar to the current state of the patient of interest (instead of using static pre-computed patient cohorts), and
4. It personalizes the treatment options by augmenting them with outcome and distributional statistics, based on retrospective analysis of the precision cohort.

Our high-level goal—to help clinicians leverage aggregated de-identified patient data for improved decision making at the point of care is—aligned with that of the “Green Button” (GB) approach^8^. The GB authors suggest that deploying a green “*patients like mine*” button as a tool in the EHR would support patient care decisions in the absence of published evidence. The GB represents personalized cohorts of retrospective observational data, constructed at the point of care, that can be analyzed to provide actionable clinical insights^32^. GB has been implemented as an informatics consult service at Stanford Medical Center^33,34^. Some key differences between our PCTO approach and the GB approach are: (1) PCTO is designed to support specific chronic diseases whereas the GB process is potentially applicable for any condition, (2) PCTO has an automated runtime to generate personalized treatment option insights in real-time whereas GB is implemented via an offline specialty consult service, (3) PCTO and GB leverage different algorithms and methods in the implementation of the main workflow components: clinical event extraction, patient similarity assessment, cohort construction, and treatment/outcome analysis.

## Methods

**Figure 1:**
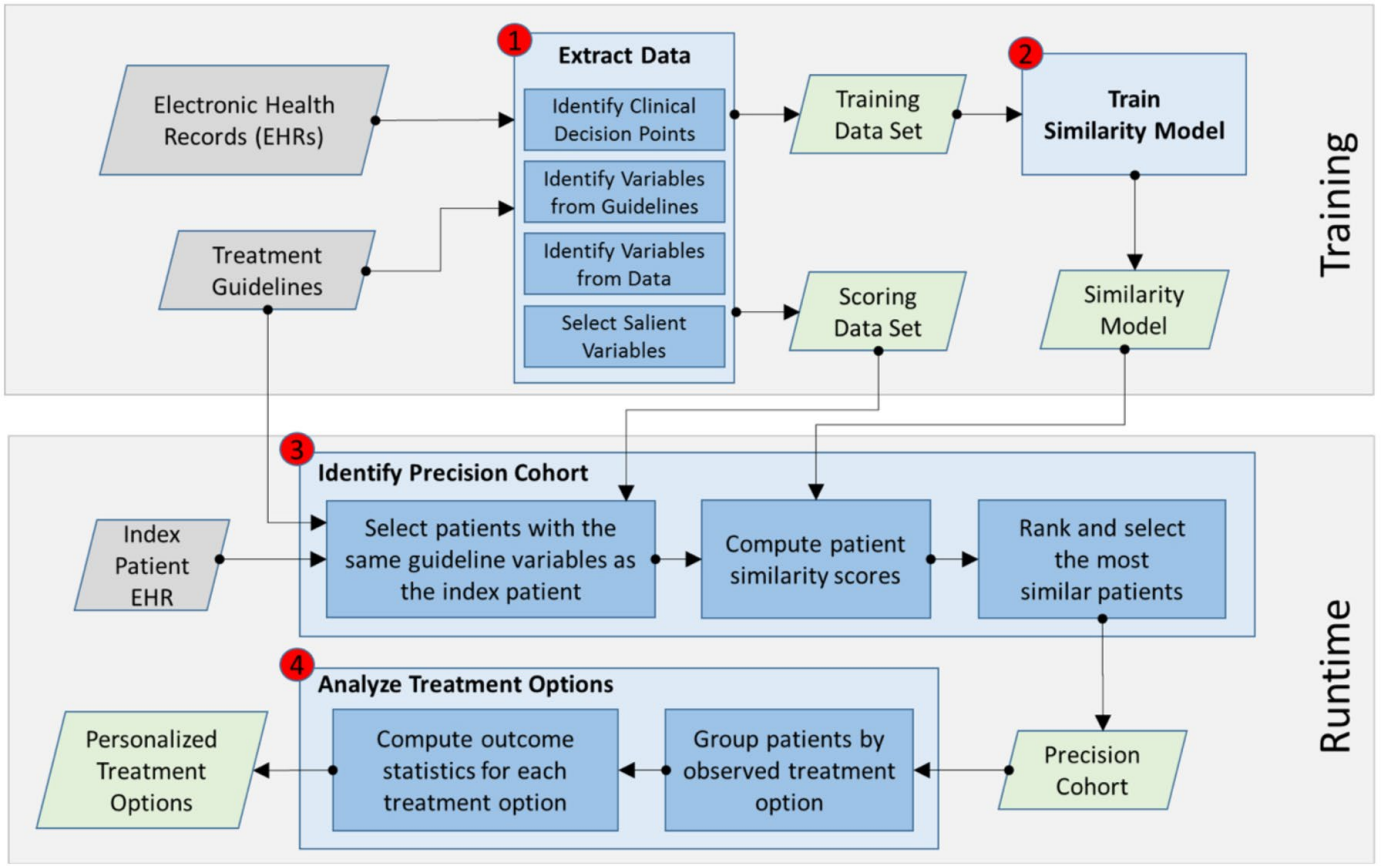
High-level Precision Cohort Treatment Options (PCTO) workflow. The PCTO workflow consists of four major steps: (1) data extraction, (2) similarity model training, (3) precision cohort identification, and 4) treatment options analysis. Steps 1 and 2 make up the training stage which is performed offline and in advance. Steps 3 and 4 form the runtime stage which is used in real-time during the patient visit encounter.

As illustrated in Fig. 1, the PCTO workflow consists of four major steps: (1) data extraction, (2) similarity model training, (3) precision cohort identification, and (4) treatment options and outcomes analysis. Steps 1 and 2 make up the training stage, which is performed offline (and in advance). Steps 3 and 4 form the runtime stage used in real-time, during the patient visit encounter. The workflow processing was implemented using a combination of SQL, Python, and R^35,36^.

### Data extraction

A four-step data extraction process was performed on the clinical treatment guidelines and EHR data, to create a training data set and a scoring data set. The training data set was used to train a model for computing patient similarity. The scoring data set served as the repository of clinical events used to create the precision cohorts at runtime. Table 1 describes each step in the data extraction process, including the method used (manual or automatic) and the domain knowledge needed (clinical or data science).

**Table 1:** Data extraction steps.

Description	Method type	Knowledge type
Identify clinical decision points. These are points in time in the patient longitudinal record where: (a) the disease is not under control, and (b) a treatment decision is warranted	Manual definition Automatic extraction	Clinical Data science
Identify variables from treatment guidelines (knowledge) associated with propensity of receiving treatment Identify relevant treatment guideline documents Identify all possible medication treatment options Identify patient characteristics used in treatment decisions	Manual	Clinical
Identify variables from electronic health records (data) Define appropriate observation window for variables Define constructor templates for different variable types Implement and run extraction on EHR data	Manual definition Automatic extraction	Clinical Data science
Select salient variables associated with outcomes of interest Identify candidates using stability ranking feature selection Manual clinical review and final selection of variables	Automatic initial passes Manual final pass	Clinical Data science

**Figure 2:**
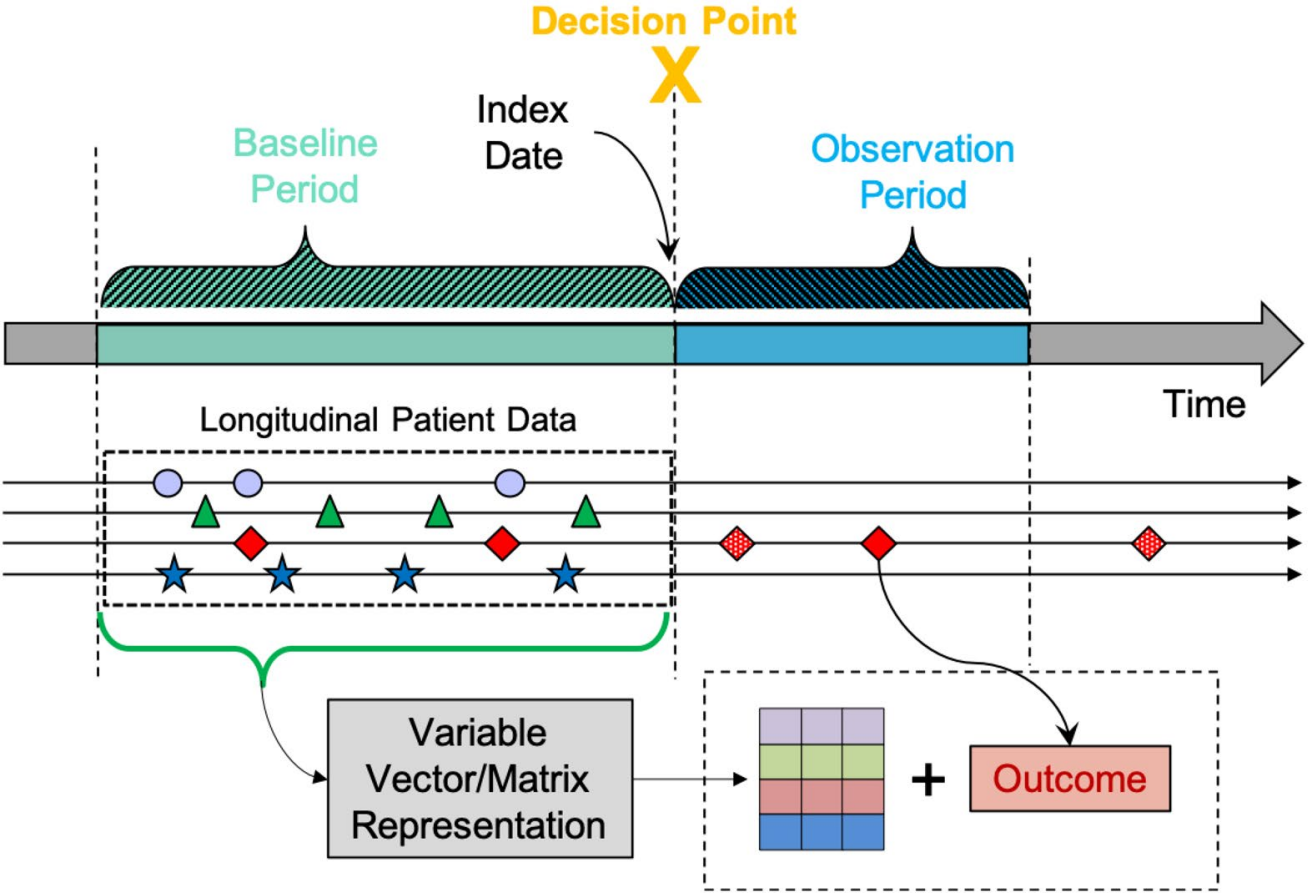
Decision point specification. Each clinical decision point is associated with an index date, a baseline period that precedes the decision point, and an observation period that follows the decision point. Data in the baseline period are processed to extract baseline variables to represent the health condition and active treatment status. Observed treatment decisions are identified at the time of the decision point. Data in the follow-up observation period are analyzed to determine the associated outcome status.

The first step was identifying and extracting appropriate points in time from the EHR, to serve as the events of interest for subsequent modeling and analysis. We identified outpatient encounters, during which a patient’s condition was not controlled, as ‘decision points’ (DPs), since they represent opportunities to initiate a change in treatment plans. The DPs were drawn from all office encounters regardless of department, and include primary care and specialist visits (refill encounters were excluded). It is common for one patient to have multiple DPs over the course of their health history. As illustrated in Fig. 2, each clinical DP is associated with a date (index date), a preceding baseline period, and an observation period that follows. Data in the baseline period were used to extract baseline variables capturing the health condition and active treatment status at the DP. Observed treatment decisions were identified at the DP, with a limited time buffer to allow for delays in treatment initiation. Data in the follow-up observation period were analyzed to determine the associated outcome status (i.e., disease controlled or uncontrolled). The selection criteria for the DPs, duration of the baseline and follow-up periods, specification of the baseline variables, identification of the treatment options, and definition of the outcomes are disease-specific and were determined by clinical expert domain knowledge. Clinical outcomes were used because of their links to important health outcomes (e.g., mortality, significant clinical events). Once all of these criteria were in place, data extraction and variable construction scripts were configured and run on the EHR data, to identify the DPs.

The second step was the manual identification of variables derived from knowledge-driven clinical guidelines. After relevant disease-specific clinical guideline documents were identified, clinical domain experts manually reviewed the published guidelines, not to extract or replicate the treatment logic, but to identify (1) the universe of recommended treatments, and (2) all of the variables used in making the treatment decisions. The recommended treatments were used to limit the treatment options considered to those that are clinically acceptable. In this work, only pharmacologic treatments were considered. Since the identified variables are associated with the propensity of receiving specific treatments, they were treated as baseline confounders for adjustment in the subsequent analysis. A subset of these variables was manually selected based on their clinical significance to be “exact match” or “filter” variables (used in the filtering step of the precision cohort construction). The remaining variables were included in the salient variable selection process (step 4).

The third step was the identification of variables from the EHR using clinical-domain-knowledge-specified variable template definitions, and implemented via automated data extraction and variable construction tools. The goal was to extract as much potentially useful information from the EHR as possible. Thousands of variables were generated by this data-driven process. Medication variables were constructed based on RxNorm concept unique identifier (RxCUI) clinical drug codes^37^. Lab variables were constructed based on Logical Observation Identifiers Names and Codes (LOINC) codes^38^. Disease variables were constructed based on the Agency for Healthcare Research and Quality’s Clinical Classifications Software (CCS) categories^39^, using codes from the International Classification of Diseases (ICD) 9th and 10th revisions^40,41^.

The fourth step was the selection of the most salient variables associated with the outcome of interest (i.e., disease control), from the pool of variables extracted from the guidelines in Step 2 and from the EHR data in Step 3. The variable selection process combined an initial automatic data-driven pass using stability ranking feature selection^42^ (to reduce the number of candidate variables) with a final manual review pass guided by clinical domain expertise. The stability selection constructed 200 different L_1_-regularized logistic regression models^43^. Each model used a random subset (75%) of the DPs and generated a set of selected features. Features that appeared consistently across many of the models were considered stable. In our implementation, only highly stable features that were selected by at least 150 of the 200 models (≥ 75%) were chosen. Data-driven correlation and clustering analysis were then performed to link highly related variables from the initial pass, to facilitate subsequent clinical review. The final manual adjudication and selection were performed, considering whether the variable was physiologically nonsensical, duplicative, or unaddressable (i.e., unmodifiable). The resulting set of variables for HTN, T2DM, and HL are described in Supplementary Tables S1, S2, and S3 online, respectively.

The result of this data extraction process was a data frame where the rows consisted of individual DPs and the columns included the treatment decision, the associated outcome, and the selected baseline variables. Only DPs with no missing variable values were retained. A random sample of the rows was selected to form the training data set and the remaining rows made up the scoring data set.

### Similarity model training

In step 2, the training data set was used to train a model for computing patient similarity. There were several challenges in training the similarity model. One challenge was that patient similarity is context dependent: two patients may be very similar to each other with respect to HTN but may differ from each other with respect to T2DM, as each disease is unique in its characteristics and etiology. The use of static similarity measures, e.g., Euclidean or Mahalanobis, for all disease conditions, may not be optimal. The similarity measure must therefore be specifically tailored to each disease. A second challenge was that the dimensionality of the variable space was very large. With all the diseases, medications, procedures, lab tests, and patient characteristics, there were thousands of variables to consider. To address these challenges, we used machine learning methods to learn the best metric from the data automatically. Specifically, we used a trainable similarity measure called locally supervised metric learning (LSML) that can be customized for a specific disease condition^12^. The method learns a disease-specific distance measure that is a generalization of the Mahalanobis distance: ${D}_{LSML}({{\varvec{x}}}_{i},{{\varvec{x}}}_{j})=\sqrt{{({{\varvec{x}}}_{i}-{{\varvec{x}}}_{j})}^{T}{\varvec{W}}{{\varvec{W}}}^{T}({{\varvec{x}}}_{i}-{{\varvec{x}}}_{j})}$, where ${{\varvec{x}}}_{i}$ and ${{\varvec{x}}}_{j}$ are the patient variable vectors for patients *i* and *j* respectively and ***W*** is a transformation weight matrix that is learned from the training data. LSML is formulated as an average neighborhood margin maximization problem and tries to find a weight matrix ***W*** where the local class discriminability (e.g., disease-controlled status) is maximized (i.e., the variable weights are determined based on their relative effect on patient outcomes). In this work, a separate LSML similarity measure was trained for each disease condition, and then used during runtime to generate the precision cohorts. The automatically learned similarity weights for each variable (computed as ${1}^{T}{\varvec{W}}{{\varvec{W}}}^{T}$) for HTN, T2DM, and HL are shown in Supplementary Tables S1, S2, and S3 online, respectively.

### Precision cohort identification

**Figure 3:**
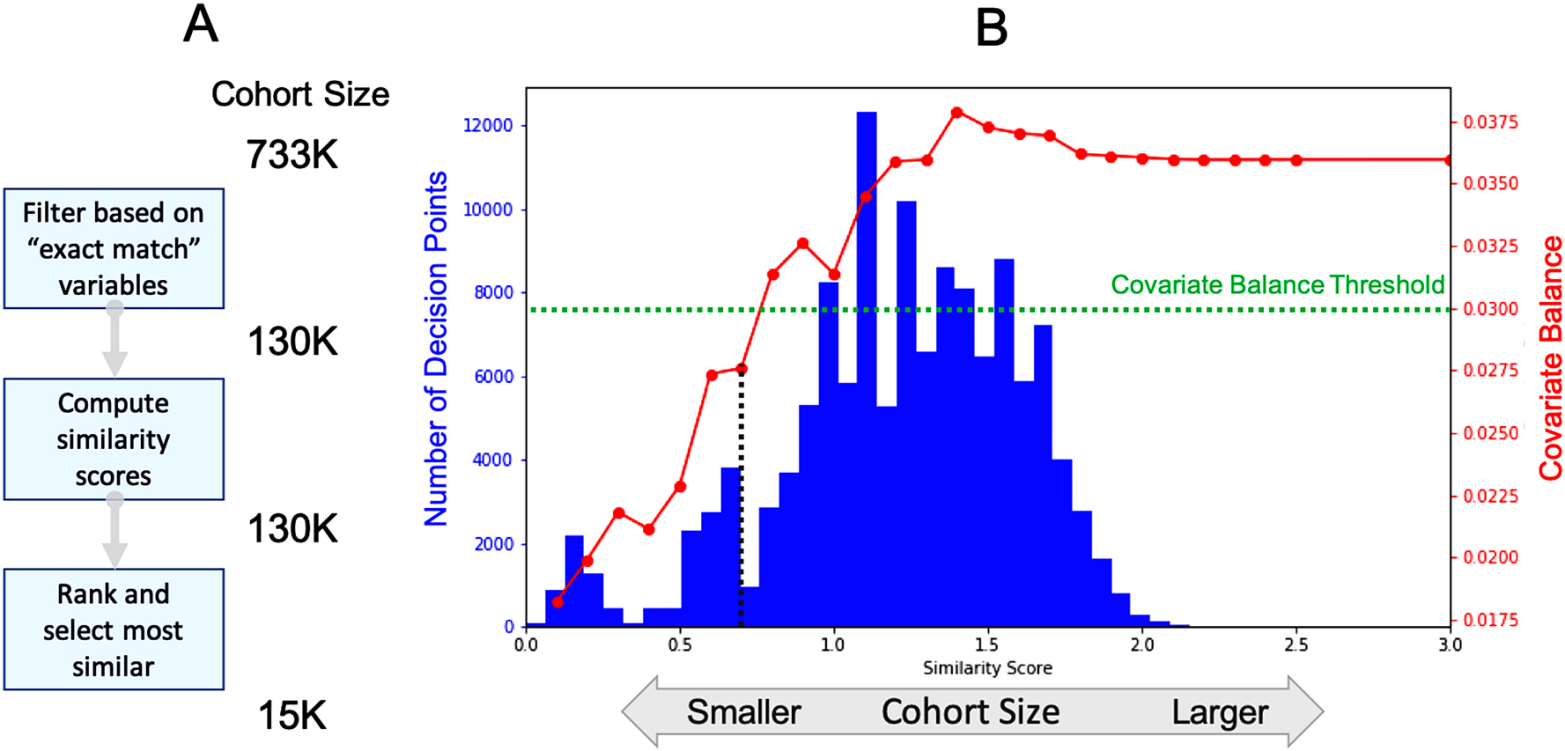
An illustration of the precision cohort construction process for one hypertension (HTN) patient of interest. (**A**) The cohort starts with all approximately 733,000 decision points in the scoring set, is reduced to approximately 130,000 after filtering based on the filter variables, and finally to approximately 15,000 after similarity scoring, ranking, and selection. (**B**) A histogram of the similarity scores for the 130,000 decision points after filtering. Those with scores closer to 0 are more similar to the patient of interest. Even when considering decision points with identical filter variables as the patient of interest, there are still many that are not very similar. Overlaid on the histogram is a plot of the covariate balance as a function of the similarity score threshold (red curve). As expected, there is a tradeoff between cohort size and covariate balance. As the cohort grows, there is more diversity, which results in less balance and higher values of the covariate balance score. A hyperparameter grid search approach can be used to find a similarity score threshold that results in a reasonably sized precision cohort with good covariate balance. In this example, a similarity score threshold of 0.7 is selected, with a covariate balance < 0.03, resulting in a precision cohort of approximately 15,000 decision points.

In step 3, a precision cohort of clinically similar patient DPs was dynamically created, at runtime, based on the data for the patient of interest at the point of care. Since the precision cohort will be used to identify the different observed treatment options, and to compare their treatment effects, the precision cohort construction process can be viewed as an approach to adjust for baseline confounders, to ensure that the treatment effect analyses are valid. Several causal inference methods can be used to adjust for baseline confounders, including matching, stratification or regression, standardization or inverse probability weighting, g-estimation, or doubly robust methods^44,45^. Although the methods have different tradeoffs in terms of capabilities and complexity^45^, when applied appropriately, any of these methods can be used to reduce bias in the treatment effect analyses for our use case. To align with how clinicians identify and select similar patient events, we used a multi-step matching based approach^46^, where the goal was to select DPs that are very similar, if not identical, to (a) one another, and (b) the current state of the patient of interest, in all respects except for one: the treatment decision at the time of the DP. The “covariate balance” of the DPs in the “no treatment change” group versus the “treatment change” group was used to assess bias and matching validity^47,48^. Covariate balance was estimated using the “standardized bias,” which was computed as the difference in means of each covariate between the two groups, divided by the standard deviation in the full treated group—smaller values closer to 0 represent better balance. The precision cohort construction process involved: (1) filtering the DPs in the scoring set, to only keep those that have identical values of the filter variables as the patient of interest, (2) computing similarity scores for the filtered DPs, using the disease-specific similarity model, and (3) ranking the DPs based on the similarity score, and selecting the most similar subset based on a tradeoff between reasonable cohort size and good covariate balance. An illustrative example of this process for one HTN patient of interest is shown in Fig. 3.

### Treatment options analysis

**Figure 4:**
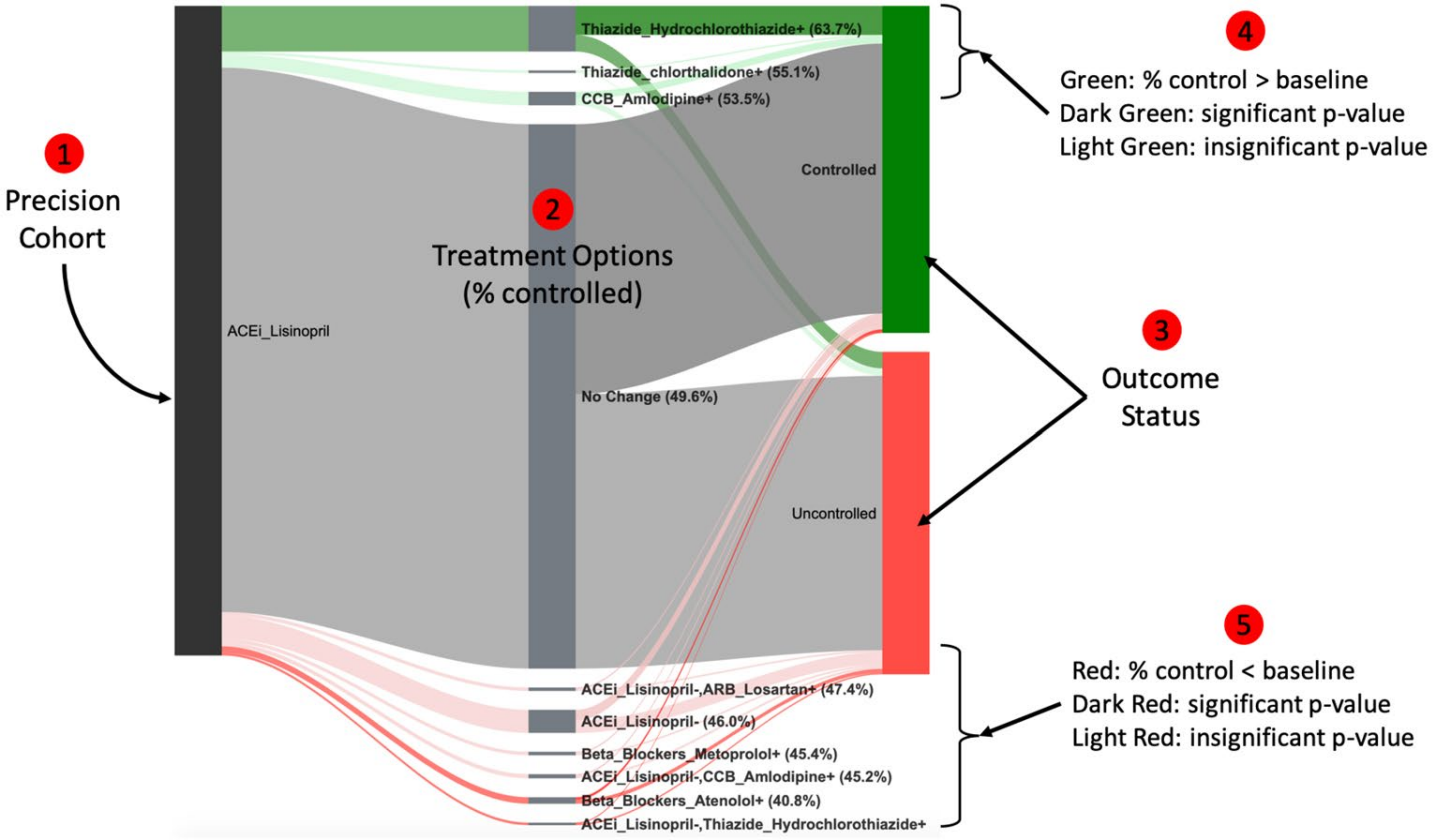
Personalized treatment options and associated outcomes in a precision cohort using a Sankey diagram. (1) The initial node (black) represents all decision points in the precision cohort for the individual patient of interest who has an HTN diagnosis, is over age 65, and is on Lisinopril (ACEI). (2) Each pathway leaving the initial node is a different treatment decision that is observed in the data. The thickness of the edge is proportional to the number of decision points. The label contains the medication class and ingredient, the start or stop of the medication (“+” or “−” suffix), and the percentage (in parentheses) of decision points under control in the follow-up. The “no change” or “stay the current course” treatment option is shown in gray and is considered the “baseline” treatment option. (3) The terminal nodes represent the outcome for the disease condition under control (green) and not under control (red). (4) Treatment options associated with better control than the baseline are shown as green pathways above the baseline with dark green indicating statistical significance. (5) Treatment options associated with worse control than the baseline are shown as red pathways below the baseline with dark red indicating statistical significance.

In step 4, a retrospective analysis was performed on the DPs in the precision cohort, to generate personalized treatment options for the individual patient of interest. This was done by first grouping the DPs by the observed treatment decision, at the time of the DP, and then, for each of these treatment option groups, computing: (1) the size of the group (number of DPs), (2) the follow-up outcome (percent controlled), (3) the difference in the outcome compared to the “no treatment change” baseline option, and (4) a statistical significance assessment of this outcome difference using a Bonferroni corrected ${\chi }^{2}$ p-value threshold of 0.05 to adjust for multiple testing^49^. The outcome comparisons for various treatment options were presented graphically to physicians, in real-time, within the EHR system, as a modified Sankey diagram^50^ (Fig. 4). As part of the clinical pilot study, we purposefully designed a visualization of the precision-cohort analysis for use by the practicing clinician at the point-of-care. We tested prototype displays through iterative feedback sessions. The final visualization appeared as a frame within the clinic’s EHR system (Supplementary Fig. S4). Because RCTs are infrequently conducted using head-to-head comparisons of treatment alternatives (new interventions are commonly tested against placebo), it is unusual for physicians to receive comparative data on medication alternatives. We found that physicians in our pilot group understood the Sankey-diagram presentation accurately.

## Results

### Study design

The PCTO workflow was applied to de-identified EHR data from a large health care provider in eastern Massachusetts, containing over 20 years of longitudinal data for approximately 2.5 million patients, for three disease conditions: HTN, T2DM, and HL. The Western Institutional Review Board (WIRB) had reviewed the study and gave it an exemption as a human subjects study. The decision-point criteria and characteristics for the three disease conditions are described in Table 2. The table lists the clinical treatment guidelines referenced, the start and end dates of the study period used for extracting data from the EHR, the decision-point criteria, the specifications for the baseline period, baseline variables, treatment options, follow-up period, and outcome. The clinical treatment guidelines were selected based on what was currently in use at the pilot health system. The study period was selected to include data covered by the time of the JNC7 publication to the present (for HTN), and the most recent 10 years of data (for T2DM and HL), so only recent and relevant treatments and practice patterns are included. The DP criteria, baseline period, follow-up period, and outcome specifications were based on clinic expert opinion. The baseline variables were derived using the process described in “Data extraction”. The treatment options were manually extracted from the guidelines, so only clinically acceptable treatments were included. Finally, the table shows the number of unique patients selected, and the number of DPs extracted based on these criteria. After the DP extraction, 50,000 DPs were selected at random to form the training data set. The remaining DPs were used as the scoring data set, following the process described in “Data extraction”. A disease-specific LSML similarity measure was trained using the training data set as described in “Similarity model training”. To perform runtime analysis, a leave-one-out cross-validation (LOOCV) approach^51^ was adopted. Each DP in the scoring data set was selected, in turn, to represent the individual patient’s office visit. That DP was then removed from the scoring data set, and the precision cohort identification (from “Precision cohort identification”) and treatment options analysis (from “Treatment Options Analysis”) were performed using the selected DP of interest and the updated scoring data set. Finally, the personalized treatment option outputs were aggregated and analyzed. The following sections present results from analyzing the data and output for individual DPs, groups of DPs, and all DPs.

**Table 2:** Decision point criteria and characteristics for the three disease conditions.

	Hypertension (HTN)	Type 2 Diabetes Mellitus (T2DM)	Hyperlipidemia (HL)
Treatment guideline	JNC-7 and JNC-8^65^	ADA^66^	AHA/ACC^53^
Study period (start–end) Rationale	1/1/2004–12/31/2018 Include data from JNC7 publication to present	1/1/2008–12/31/2018 Use only the most recent 10 years of data	1/1/2008–12/31/2018 Use only the most recent 10 years of data
Decision point (DP) criteria	Identify all two consecutive uncontrolled BP readings (SBP ≥ 140 or DBP ≥ 90 mmHg) separated by 1 to 365 days The date of the second encounter is a decision point Age ≥ 18 at DP Not pregnant in prior 12 months	If the patient has a T2DM diagnosis on the problem list, the index date is the date of the diagnosis. If the patient does not have a T2DM problem list diagnosis, find the earliest two consecutive HbA1c ≥ 6.5% readings separated by 1 to 365 days, and the index date is the date of the second encounter All encounters after the index date with an associated HbA1c ≥ 7.0% within 365 days prior to the encounter date is a decision point Age ≥ 18 at DP Not pregnant in prior 12 months	The patient must have an HL diagnosis on the problem list All encounters after the HL diagnosis with an associated LDL > 130 mg/dl within 420 days prior to the encounter date is a decision point Age ≥ 18 at DP Not pregnant in prior 12 months
Baseline period	The start of the baseline period depends on the specific variable. Some variables use data within the past 12 months, past 14 months, or all available past history. The end of the baseline period is the decision point date		
Baseline variables	The variable selection process described in “Data extraction” was performed. The final set of selected variables for HTN, T2DM, and HL are described in Supplementary Tables S1, S2, and S3, online respectively		
Treatment options	The list of clinically acceptable medication treatments was obtained via manual review of the relevant clinical treatment guidelines. The treatment variables for HTN, T2DM, and HL can be found in Supplementary Tables S1, S2, and S3, online respectively		
Follow-up period	1–365 or 14–365 days after the decision point date	90–365 days after the decision point date	30–450 days after the decision point date
Outcome specification	The first BP between N and 365 days after the decision point date. If no treatment changed, N = 0, otherwise N = 14. An SBP ≥ 140 or DBP ≥ 90 mmHg is considered not controlled	The first HbA1c lab test result between 90 and 365 days after the decision point date. An HbA1c ≥ 7% is considered not controlled	The first LDL lab test result between 30 and 450 days after the decision point date. An LDL > 130 mg/dl is considered not controlled
Number of patients	157,942	24,373	63,510
Number of decision points	733,300	171,203	561,971

*DP* decision point, *BP* blood pressure, *DBP* diastolic blood pressure, *SBP* systolic blood pressure, *LDL* low density lipoprotein, *HbA1c* hemoglobin A1C.

In terms of computational cost, the extraction of the data to create the training and scoring data sets typically took under one processor-hour. Creating a new scoring model took between 4 to 8 processor-hours, and the dynamic generation of the precision cohort and personalized treatment options report for an individual patient of interest took a few processor-seconds. The entire LOOCV analysis typically took 3000 processor-hours (since all DPs had to be processed). However, since the runtime was parallelizable, the analysis was performed on a cluster of approximately 160 processors, thus providing results within a day.

### Filter variable cohorts

The largest observed cohorts based on the “exact match” filter variables for HTN, T2DM, and HL are shown in Supplementary Figs. S5, S6, and S7, online, respectively. Since the filter variables capture the active treatments and key patient characteristics used to inform treatment decisions (based on the clinical guidelines), these cohorts represent the most commonly observed active treatment combinations at the DPs. Although there is a large number of cohorts (32,656 for HTN, 1867 for T2DM, and 253 for HL), it is much less than all the possible combinations (2^90^ for HTN, 2^48^ for T2DM, and 2^14^ for HL); moreover the cumulative coverage curve indicates that most DPs belong to only a small number of cohorts (40% in the top 10 cohorts for HTN, 62% for T2DM, and 91% for HL) and that there is a long tail (70% of the groups have less than five DPs for HTN, 60% for T2DM, and 34% for HL). These observations are consistent with other published findings where the number of unique treatment pathways for HTN and T2D is large and diverse^52^. Unsurprisingly, the largest cohorts are those that are not currently on any treatment, followed by cohorts that are only on a single treatment (e.g., Lisinopril for HTN, Metformin for T2DM, and Simvastatin for HL). For HTN and T2D, the size of the “no treatment” cohorts may be increased because the selection criteria (Table 2) can include DPs where the patient does not have an official disease diagnosis (i.e., based only on abnormal lab test measurements). It is also possible that some diagnosed patients are only on lifestyle (e.g., diet and exercise) modification treatments that are not explicitly tracked in the analysis. Although an explicit disease diagnosis is required for HL, there is variability in the initiation of pharmacologic treatment based on LDL levels, which results in more “no treatment” DPs (especially for younger patients and those with borderline LDL levels)^53^. In addition, similar to HTN and T2D, some HL patients may only be on non-medication lifestyle modification treatments.

### Global treatment options

The largest observed global treatment option decisions across all DPs for HTN, T2DM, and HL are shown in Supplementary Figs. S8, S9, and S10 online, respectively. Across all three conditions, the most common treatment decision is to “stay the current course” and make no change to the current medication-treatment ingredient (81% for HTN, 85% for T2DM, and 94% for HL). Note that since the dosage was not considered, treatment decisions that only changed the dosage appeared as refills and were considered “no ingredient change.” For HTN and T2DM, some no-change treatments reflect no active pharmacologic treatments for undiagnosed patients. Even for undiagnosed patients who have measurement-proven conditions, however, “clinical inertia” occurs when a PCP does not treat a condition that is not under control. Due to the large number of DPs without any active treatments, the next most common set of treatment option decisions are the start of first-line medications: Lisinopril (ACEI) and Hydrochlorothiazide (thiazide diuretic) for HTN, Metformin (biguanides) for T2DM, and Simvastatin and Atorvastatin (statin) for HL. Overall, there are many treatment option decisions (3966 for HTN, 883 for T2DM, and 223 for HL). However, many are unique and have only a single DP (60% for HTN, 53% for T2DM, and 29% for HL), again consistent with prior findings on the uniqueness of treatment pathways^52^. In terms of treatment decisions that switch ingredients within the same class, it is interesting to note that, for T2DM, the most common are Sulfonylurea changes: stopping Glyburide and starting Glipizide or Glimepiride. For HL, the most common are Statin changes: stopping Simvastatin and starting Atorvastatin, Pravastatin, or Rosuvastatin.

### Personalized treatment options

Figure 4 illustrates the patient-level personalized treatment options and associated outcomes, resulting from applying the PCTO analysis to a single DP for an individual patient. In this case, the patient has an HTN diagnosis, is over age 65, and is on Lisinopril (ACEI) at the time of the DP. In addition to identifying treatment options that have statistically significantly better outcomes (dark green pathways) than the baseline “no change” treatment option (grey pathway), the analysis also lists treatment options that have significantly worse outcomes (dark red pathways). Options in light green or light red have better or worse outcomes, respectively, but are not statistically significant. The complexity of each treatment option, estimated as the number of unique medications, is also available.

**Figure 5:**
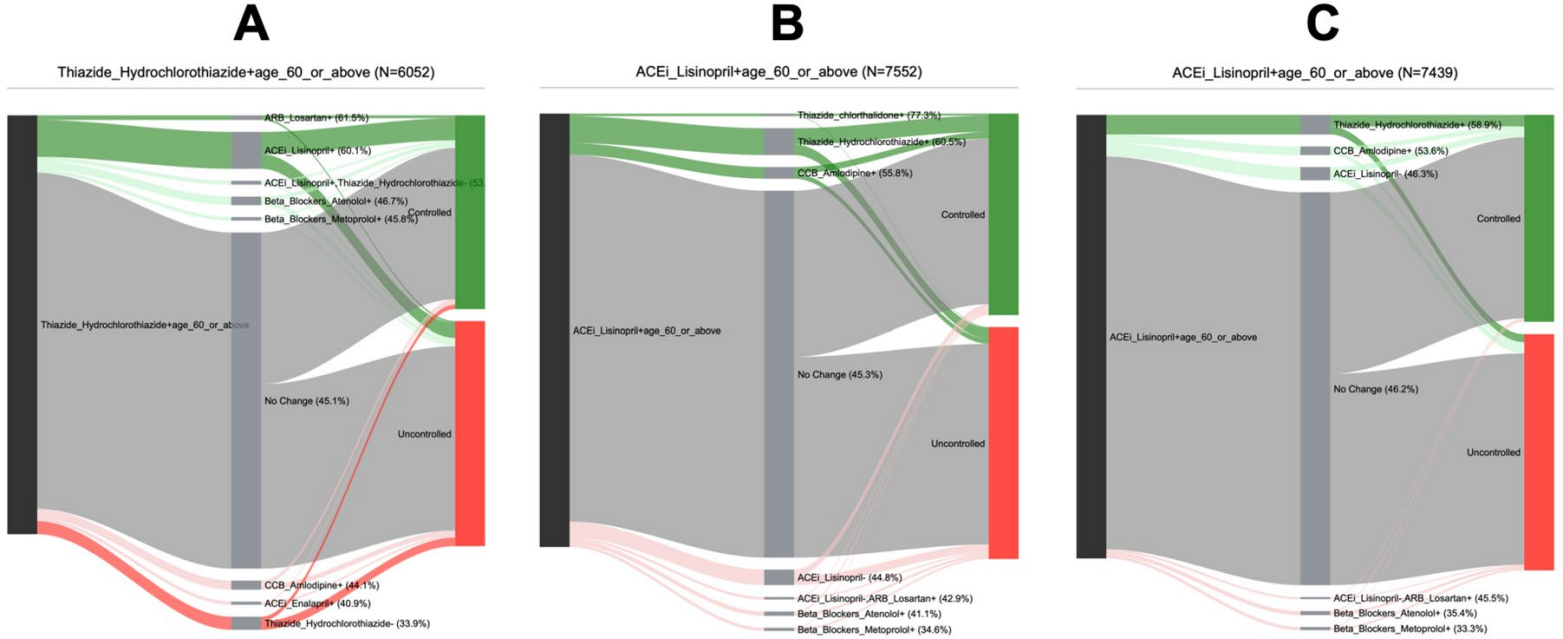
Personalized treatment options for three different HTN patient decision points. (**A**) The patient decision point belongs to a filter variable group that is over age 60 and on Hydrochlorothiazide (Thiazide). (**B**, **C**) The patient decision point belongs to a filter variable group that is over age 60 and on Lisinopril (ACEI). Note that the treatment options associated with significantly better control (dark green pathways) across the three patient decision points are different.

DPs from different filter variable cohorts are expected to have different personalized treatment options. For example, Fig. 5A shows the treatment options for a DP, in a group that is over age 60, and on Hydrochlorothiazide (Thiazide), while Fig. 5B shows the treatment options for a DP, in a group that is over age 60, and on Lisinopril (ACEI). The treatment options associated with significantly better-than-the-baseline outcomes (dark green pathways), for these two DPs, are different: in Fig. 5A, it is adding Lisinopril (ACEI), while in Fig. 5B, it is adding either Amlodipine (CCB) or a Thiazide (Hydrochlorothiazide or Chlorthalidone). However, patient DPs that belong to the same filter variable cohort can also have different personalized treatment options. Figure 5C shows the personalized treatment options for a patient DP that belongs to the same group as Fig. 5B [over age 60 and on Lisinopril (ACEI)], but the effective treatment options here are different: only adding Hydrochlorothiazide passes the selection criteria. Since the precision cohort is created dynamically for each patient DP, based on its specific characteristics, the composition of the precision cohort can differ, resulting in different personalized treatment options.

To assess the applicability and potential impact of the PCTO method, and to better understand its behavioral characteristics, all DPs in the scoring data set were processed using the LOOCV strategy described in “Results”; the results are summarized in Supplementary Figs. S11, S12, and S13 online (for HTN, T2DM, and HL, respectively). Each figure shows the number of DPs, grouped by filter variable cohorts (rows), and the percentage of DPs that have personalized treatment options with statistically significantly better outcome (green), with fewer medications (purple), with the same number medications (yellow), and with more medications (red). As expected, filter variable cohorts with no active medication treatments (e.g., “All guideline variables false” for all conditions, “age_60_or_above” for HTN, “T2D” for HL) have the largest percentage with significantly better treatment options. For HTN, it is interesting to note that for the African American (“black”) group on no active HTN medications, only 70% of the DPs have treatment options with statistically significantly better outcomes compared to taking no HTN medications. Similar behavior is seen in the diabetes (“dm”) group on no active HTN medications where only 78% of the DPs have treatment options with statistically significantly better outcomes. This implies that it may be more challenging to control HTN for patients that are black or have diabetes and is consistent with prior findings^54,55^. With a few exceptions, filter variable cohorts with one active medication treatment (e.g., “ACEI_Lisinopril” or “Thiazide_Hydrochlorothiazide” for HTN, “Biguanides_Metformin” and “Sulfonylurea_Glyburide” for T2DM, and “Statin_Simvastatin” and “Statin_Pravastatin” for HL) have the next largest percentage with significantly better treatment options. The notable exceptions are “Beta_Blockers_Metoprolol” for HTN, “Insulin_Glargine” for T2DM, and “Statin_Atorvastatin” and “Statin_Rosuvastatin” for HL. Indeed, beta blockers for HTN and insulin for T2DM are not typically first-line treatments, and patients on these medications likely have already tried (unsuccessfully) other medications before settling on these. For HL, atorvastatin and rosuvastatin are the most effective statins available^56^ and would be difficult to improve upon. Recognizing that successful disease management should not only consider disease control, but other factors such as treatment complexity and cost^57^, information about the number of medications relative to the baseline (fewer, same, more) for each treatment option is also computed. Across the three diseases, many filter variable cohorts that are on multiple medications, have treatment options with fewer or the same number of medications with comparable outcomes (percent controlled). Treatment options with more medications are almost always possible. The impact and utility of PCTO are dependent on the specific filter variable cohort, but overall, 75%, 74%, and 85% of all the DPs for HTN, T2DM, and HL, respectively, have at least one significantly better treatment option identified.

## Discussion

Physicians are encouraged to base treatment decisions on clinical guidelines, where available. Unfortunately, the evidence basis for clinical guidelines relies on the results of RCTs whose inclusion/exclusion criteria exclude the majority of the population. That makes it difficult to appropriately extrapolate the experience from clinical trials to the general population, or to individual patients. Similarly, generating an analysis of undifferentiated observational data derived from the experiences of the general population, would not lead to insights sufficiently relevant to a particular patient.

We developed an analytic workflow to create precision cohorts representing patients similar to the patient of interest, using a model derived from machine learning. Tracking and analyzing the clinical responses of similar patients in similar clinical situations, when prescribed various medication treatments, provides physicians with observational data from real-world experience as input to inform decisions about an individual patient. Our analysis of historical observational data does not lead to recommended actions, nor does it replace clinical guidelines. These analyses supplement guidelines based on RCTs and expert opinion and require human judgment to appropriately incorporate the results of the statistical analysis into medical decision making.

The PCTO workflow, summarized in Fig. 1, can be applied to any disease condition, as long as the necessary data and criteria are provided. For the results presented in “Results”, the specific criteria were finalized after several iterations of a specification, implementation, analysis, and review process involving both data scientists and clinicians, and represent just one set of clinically relevant use cases for HTN, T2DM, and HL.

There are several limitations to the current work. Due to data availability, only medication-based treatment options were considered, and dosage information was not available. As mentioned in “Results”, this limited the granularity of the analysis: non-pharmacologic treatments would appear as “no treatment” and changes only in drug dose would appear as “no change in drug class.” Since nothing in the method explicitly precludes handling additional treatment types, a natural extension would be to include non-pharmacologic treatment options such as diet, exercise, smoking cessation, and other lifestyle modifications or interventions, to ameliorate unfavorable social determinants of health (SDOH), as long as the necessary data is available. Dosage information, if available, could also be included to differentiate the treatment options.

The study only explored three chronic conditions: HTN, T2DM, and HL. The method should be applied to other common chronic disease conditions (e.g., chronic obstructive pulmonary disease and congestive heart failure) to better understand how the method can generalize.

The patient characteristics used to create the precision cohorts in the pilot were limited to information available in the outpatient EHR. More data from external sources could potentially help refine cohort measurement, making it even more precise. Given appropriate data, for example, variables could be included to capture social, behavioral, and adherence factors that have been shown to have a significant impact on health outcomes^58,59^.

The method is sensitive to the size of the precision cohort. Our data set contained approximately 2.5 million patients from one health system. Although the size of our patient pool was sufficient to provide statistically significant treatment options for the common diseases we studied, less common diseases may not allow us to identify statistically meaningful precision cohorts. Combining data from multiple health systems would increase the numbers but may introduce other confounders that influence the similarity of patients. Applying our methods to data from other health systems would help assess the generalizability of our approach. Another approach is to make more efficient use of the available data by using all of the DPs, by weighting their contribution based on the similarity score, instead of thresholding and using only a small subset.

There are additional limitations to the use of observational data. In addition to missingness, incompleteness, and errors, there is increasing attention to the inadvertent presence of bias in training data^60^. Even with the causal inference mitigation methods described in “Precision cohort identification”, the selection of confounder covariates may not be fully complete, and biases may not be eliminated entirely. In this work, we used observational data from EHRs, which has rich clinical information (such as lab test results) that was important for our use case of being able to assess chronic disease control. Other types of observational healthcare data, such as claims, have not been found to be as reliable^61^. However, claims data can be leveraged for different use cases, where less detailed information is required, e.g., if the outcomes can be characterized using only billing codes^62^.

Finally, this work used data from a single healthcare system, and the DPs were extracted from historical data, which may contain outdated treatment information and historical biases^63^. We attempted to mitigate the impact of this by adjusting the lookback period to coincide with the publication date of the most recent national guidelines and only considering drugs approved by current guidelines. However, prospective evaluation and independent validation of the PCTO workflow on data from different healthcare systems are needed to establish and verify its clinical utility and effectiveness on patient health outcomes.

In addition to addressing these limitations in future work, there are other possible extensions. One is to leverage the method to perform retrospective population-based analysis that could be useful to both healthcare providers and payers. The current approach to population health management relies on creating reports of patients whose disease was not under control when the report was generated. From a patient-risk perspective, this time-slice view does not adequately reflect the cumulative morbidity risks of patients’ uncontrolled state over time. From a provider perspective, the periodic reports do not portray the opportunities the health professional had to make changes to the interventions to improve the control of the chronic condition. One possible approach to address these issues is to apply the PCTO workflow to historical patient data, compare the resulting personalized treatment options and outcomes against the actual treatment options and outcomes, analyze the data to identify potential care gaps and missed treatment decision opportunities, and then aggregate the results across the DPs to generate population-level insights. We recently performed this “precision population analysis” and found that, for the majority of DPs, there were alternative treatment options administered to patients in the precision cohort that resulted in a significantly increased proportion of patients under control than the actual treatment option chosen for the index patient^64^.

In summary, we presented a novel approach to analyzing observational data from a large number of patients to derive best practices that achieve better clinical outcomes for similar patients. The workflow was successfully implemented using EHR data from a large health care provider for three different chronic diseases (HTN, T2DM, and HL). A retrospective analysis demonstrated that alternative treatment options with better outcomes were available for a majority of cases. The resultant models for HTN and T2DM were deployed in a pilot study with PCPs who used it during clinic visits. Presenting AI-derived insight from analysis of population data in the EHR can complement recommendations from clinical guidelines and personal experience, to help physicians personalize treatment decisions for individual patients.

## Supplementary Information

Supplementary Information.

## Data Availability

The data that support the findings of this study are available from Atrius Health, but restrictions apply to the availability of these data, which were used under license for the current study, and so are not publicly available. Data are however available from the authors upon reasonable request and with permission of Atrius Health.

## Abbreviations

ACEI: Angiotensin converting enzyme inhibitor
ACC: American college of cardiology
ADA: American diabetes association
AHA: American heart association
CCB: Calcium channel blocker
CCS: Clinical classifications software
CDS: Clinical decision support
CKD: Chronic kidney disease
EHR: Electronic health record
GB: Green Button
HL: Hyperlipidemia
HTN: Hypertension
ICD: International classification of diseases
JNC: Joint national committee
LOOCV: Leave-one-out cross-validation
LSML: Locally supervised metric learning
PCP: Primary care physician
PCTO: Precision cohort treatment options
RCT: Randomized clinical trial
RxCUI: RxNorm concept unique identifier
SDOH: Social determinants of health
T2DM: Type 2 diabetes mellitus
