# Supplementary material for: Personalized treatment options for chronic diseases using precision cohort analytics
Source: Sci Rep. 2021 Jan 13;11:1139. doi: 10.1038/s41598-021-80967-5 (PMC7806725; doi:10.1038/s41598-021-80967-5)
Supplement: Supplementary file 1 — Supplementary Information. [file 41598_2021_80967_MOESM1_ESM.pdf]

SUPPLEMENTARY INFORMATION

Title:
Personalized Treatment Options for Chronic Diseases using Precision Cohort Analytics

Authors:
Kenney Ng[a], Uri Kartoun[a], Harry Stavropoulos[a], John A. Zambrano[b], Paul C. Tang[c]

Author Affiliations:
[a]Center for Computational Health, IBM Research, Cambridge, MA, USA
[b]Atrius Health, Boston, MA, USA
[c]Stanford Clinical Excellence Research Center, Stanford, CA, USA

N	Variable Name	Use	Source	Type	Description	Similarity Weight
1	DATA_age	Similarity	Data	Continuous	Age of patient on the index date	0.0345
2	DATA_count_14mo_A1C	Similarity	Data	Continuous	Number of hemoglobin A1C (HbA1c) lab tests in the past 14 months (from index date)	0.0396
3	DATA_count_14mo_cbc_panel	Similarity	Data	Continuous	Number of complete blood count (CBC) panel lab tests in the past 14 months (from index date)	0.1680
4	DATA_count_14mo_electrolyte_panel	Similarity	Data	Continuous	Number of electrolyte panel lab tests in the past 14 months (from index date)	0.2429
5	DATA_count_14mo_INR	Similarity	Data	Continuous	Number of prothrombin time international normalized ratio (INR) lab tests in the past 14 months (from index date)	0.1009
6	DATA_count_14mo_lipid_panel	Similarity	Data	Continuous	Number of lipid panel lab tests in the past 14 months (from index date)	0.3673
7	DATA_count_14mo_Preceding_ER_Visits	Similarity	Data	Continuous	Number of emergency room/department encounters in the past 14 months (from index date)	0.0114
8	DATA_count_14mo_Preceding_Office_Visits_or_TELEPHONE_or_EMAIL	Similarity	Data	Continuous	Number of office visit, telephone, or email encounters in the past 14 months (from index date)	1.0046
9	DATA_count_antihypertensive_ACTIVE_meds	Similarity	Data	Continuous	Number of active antihypertensive medication prescriptions on the index date	1.9374
10	DATA_is_alcohol_use	Similarity	Data	Boolean	Did the patient respond yes to the "uses alcohol" lifestyle question before the index date?	0.1133
11	DATA_is_Asthma	Similarity	Data	Boolean	Is the patient diagnosed with asthma before the index date?	0.6541
12	DATA_is_Back_problem	Similarity	Data	Boolean	Is the patient diagnosed with back problems before the index date?	0.0815
13	DATA_is_Coron_athero	Similarity	Data	Boolean	Is the patient diagnosed with coronary arthoscerlosis before the index date?	0.6792
14	DATA_is_Depression_disorder	Similarity	Data	Boolean	Is the patient diagnosed with depression disorder before the index date?	0.2481
15	DATA_is_Headache_mig	Similarity	Data	Boolean	Is the patient diagnosed with migrane headaches before the index date?	0.4987
16	DATA_is_HTN	Similarity	Data	Boolean	Is the patient diagnosed with hypertension before the index date?	0.6538
17	DATA_is_most_recent_Hematocrit_Normal	Similarity	Data	Boolean	Is the most recent (within 12 months) hematocrit lab test normal?	0.2816
18	DATA_PROC_Flu_vaccine_15mo	Similarity	Data	Boolean	Did the patient complete a flu vaccine in the past 15 months (from index date)	0.5537
19	KNOWLEDGE_is_age_60_or_above_AND_is_PROC_Colonoscopy	Similarity	Knowledge	Boolean	Is patient age >=60 on the index date and completed a colonoscopy procedure within the past 11 years?	0.5163
20	KNOWLEDGE_is_hyperlipidemia_AND_is_most_recent_LDL_Cholesterol_NormalorLow	Similarity	Knowledge	Boolean	Is patient diagnosed with hyperlipidemia before the index date and is the most recent (within 12 months) low density lipid (LDL) test normal or low?	1.1299
21	GUIDELINES__DBP_Value_at_Index_Date	Similarity	Knowledge	Continuous	Diastolic Blood Pressure value on the index date	0.5059
22	GUIDELINES__SBP_Value_at_Index_Date	Similarity	Knowledge	Continuous	Systolic Blood Pressure value on the index date	0.7196
23	GUIDELINES__is_DBP_Value_at_Index_Date_Under_Control	Similarity	Knowledge	Boolean	Is the diastolic blood pressure under control (<90) on the index date?	1.2973
24	GUIDELINES__is_SBP_Value_at_Index_Date_Under_Control	Similarity	Knowledge	Boolean	Is the systolic blood pressure under control (<140) on the index date?	0.9694
25	GUIDELINES__is_age_60_or_above	Filter	Knowledge	Boolean	Is the patient age >= 60 on the index date?	1.0176
26	GUIDELINES__is_black	Filter	Knowledge	Boolean	Is the patient's race black?	0.3859
27	GUIDELINES__is_CAD_Established	Filter	Knowledge	Boolean	Is the patient diagnosed with established coronary artery disease (CAD) before the index date?	0.4418
28	GUIDELINES__is_CAD_Risk	Filter	Knowledge	Boolean	Is the patient diagnosed with risk of coronary artery disease (CAD) before the index date?	0.7381
29	GUIDELINES__is_ckd	Filter	Knowledge	Boolean	Is the patient diagnosed with chronic kidney disease (CKD) before the index date?	0.7262
30	GUIDELINES__is_dm	Filter	Knowledge	Boolean	Is the patient diagnosed with diabetes before the index date ?	0.3306
31	GUIDELINES__is_HF_LVD	Filter	Knowledge	Boolean	Is the patient diagnosed with heart failure (HF) or left ventricular dysfunction (LVD) before the index date?	1.1174
32	GUIDELINES_is_ACEi_Benazepril_ACTIVE	Filter	Knowledge	Boolean	Does the patient have an active Benazepril angiotensin-converting-enzyme inhibitor (ACEI) prescription on the index date?	0.1820
33	GUIDELINES_is_ACEi_Captopril_ACTIVE	Filter	Knowledge	Boolean	Does the patient have an active Captopril angiotensin-converting-enzyme inhibitor (ACEI) prescription on the index date?	0.0287
34	GUIDELINES_is_ACEi_Enalapril_ACTIVE	Filter	Knowledge	Boolean	Does the patient have an active Enalapril angiotensin-converting-enzyme inhibitor (ACEI) prescription on the index date?	0.3987
35	GUIDELINES_is_ACEi_Enalaprilat_ACTIVE	Filter	Knowledge	Boolean	Does the patient have an active Enalaprilat angiotensin-converting-enzyme inhibitor (ACEI) prescription on the index date?	1.2092
36	GUIDELINES_is_ACEi_Fosinopril_ACTIVE	Filter	Knowledge	Boolean	Does the patient have an active Fosinopril angiotensin-converting-enzyme inhibitor (ACEI) prescription on the index date?	0.3487
37	GUIDELINES_is_ACEi_Lisinopril_ACTIVE	Filter	Knowledge	Boolean	Does the patient have an active Lisinopril angiotensin-converting-enzyme inhibitor (ACEI) prescription on the index date?	0.2215
38	GUIDELINES_is_ACEi_Moexipril_ACTIVE	Filter	Knowledge	Boolean	Does the patient have an active Moexipril angiotensin-converting-enzyme inhibitor (ACEI) prescription on the index date?	0.9657
39	GUIDELINES_is_ACEi_Perindopril_ACTIVE	Filter	Knowledge	Boolean	Does the patient have an active Perindopril angiotensin-converting-enzyme inhibitor (ACEI) prescription on the index date?	0.8212
40	GUIDELINES_is_ACEi_Quinapril_ACTIVE	Filter	Knowledge	Boolean	Does the patient have an active Quinapril angiotensin-converting-enzyme inhibitor (ACEI) prescription on the index date?	0.2940
41	GUIDELINES_is_ACEi_Ramipril_ACTIVE	Filter	Knowledge	Boolean	Does the patient have an active Ramipril angiotensin-converting-enzyme inhibitor (ACEI) prescription on the index date?	0.3510
42	GUIDELINES_is_ACEi_Trandolapril_ACTIVE	Filter	Knowledge	Boolean	Does the patient have an active Trandolapril angiotensin-converting-enzyme inhibitor (ACEI) prescription on the index date?	0.1524
43	GUIDELINES_is_Aldosterone_Receptor_Blocker_Eplerenone_ACTIVE	Filter	Knowledge	Boolean	Does the patient have an active Eplerenone aldosterone receptor blocker prescription on the index date?	0.4543
44	GUIDELINES_is_Aldosterone_Receptor_Blocker_Spironolactone_ACTIVE	Filter	Knowledge	Boolean	Does the patient have an active Spironolactone aldosterone receptor blocker prescription on the index date?	0.8213
45	GUIDELINES_is_Alpha_Blocker_Carvedilol_ACTIVE	Filter	Knowledge	Boolean	Does the patient have an active Carvedilol alpha blocker prescription on the index date?	0.0849
46	GUIDELINES_is_Alpha_Blocker_Doxazosin_ACTIVE	Filter	Knowledge	Boolean	Does the patient have an active Doxazosin alpha blocker prescription on the index date?	0.4450
47	GUIDELINES_is_Alpha_Blocker_Labetalol_ACTIVE	Filter	Knowledge	Boolean	Does the patient have an active Labetalol alpha blocker prescription on the index date?	0.2108
48	GUIDELINES_is_Alpha_Blocker_Phenoxybenzamine_ACTIVE	Filter	Knowledge	Boolean	Does the patient have an active Phenoxybenzamine alpha blocker prescription on the index date?	0.2712
49	GUIDELINES_is_Alpha_Blocker_Phentolamine_ACTIVE	Filter	Knowledge	Boolean	Does the patient have an active Phentolamine alpha blocker prescription on the index date?	0.2617
50	GUIDELINES_is_Alpha_Blocker_Prazosin_ACTIVE	Filter	Knowledge	Boolean	Does the patient have an active Prazosin alpha blocker prescription on the index date?	0.0391
51	GUIDELINES_is_Alpha_Blocker_Terazosin_ACTIVE	Filter	Knowledge	Boolean	Does the patient have an active Terazosin alpha blocker prescription on the index date?	0.4743
52	GUIDELINES_is_ARB_Azilsartan_ACTIVE	Filter	Knowledge	Boolean	Does the patient have an active Azilsartan angiotensin receptor blocker (ARB) prescription on the index date?	0.0628
53	GUIDELINES_is_ARB_Candesartan_ACTIVE	Filter	Knowledge	Boolean	Does the patient have an active Candesartan angiotensin receptor blocker (ARB) prescription on the index date?	0.0404
54	GUIDELINES_is_ARB_Eprosartan_ACTIVE	Filter	Knowledge	Boolean	Does the patient have an active Eprosartan angiotensin receptor blocker (ARB) prescription on the index date?	1.2216
55	GUIDELINES_is_ARB_Irbesartan_ACTIVE	Filter	Knowledge	Boolean	Does the patient have an active Irbesartan angiotensin receptor blocker (ARB) prescription on the index date?	0.5319
56	GUIDELINES_is_ARB_Losartan_ACTIVE	Filter	Knowledge	Boolean	Does the patient have an active Losartan angiotensin receptor blocker (ARB) prescription on the index date?	0.1374
57	GUIDELINES_is_ARB_Olmesartan_ACTIVE	Filter	Knowledge	Boolean	Does the patient have an active Olmesartan angiotensin receptor blocker (ARB) prescription on the index date?	0.4190
58	GUIDELINES_is_ARB_Telmisartan_ACTIVE	Filter	Knowledge	Boolean	Does the patient have an active Telmisartan angiotensin receptor blocker (ARB) prescription on the index date?	0.1022
59	GUIDELINES_is_ARB_Valsartan_ACTIVE	Filter	Knowledge	Boolean	Does the patient have an active Valsartan angiotensin receptor blocker (ARB) prescription on the index date?	0.4698
60	GUIDELINES_is_Beta_Blockers_Acebutolol_ACTIVE	Filter	Knowledge	Boolean	Does the patient have an active Acebutolol beta blocker prescription on the index date?	0.1076
61	GUIDELINES_is_Beta_Blockers_Atenolol_ACTIVE	Filter	Knowledge	Boolean	Does the patient have an active Atenolol beta blocker prescription on the index date?	1.2831
62	GUIDELINES_is_Beta_Blockers_Betaxolol_ACTIVE	Filter	Knowledge	Boolean	Does the patient have an active Betaxolol beta blocker prescription on the index date?	0.5661
63	GUIDELINES_is_Beta_Blockers_Bisoprolol_ACTIVE	Filter	Knowledge	Boolean	Does the patient have an active Bisoprolol beta blocker prescription on the index date?	0.2948
64	GUIDELINES_is_Beta_Blockers_Carvedilol_ACTIVE	Filter	Knowledge	Boolean	Does the patient have an active Carvedilol beta blocker prescription on the index date?	0.5376
65	GUIDELINES_is_Beta_Blockers_Labetalol_ACTIVE	Filter	Knowledge	Boolean	Does the patient have an active Labetalol beta blocker prescription on the index date?	1.0247
66	GUIDELINES_is_Beta_Blockers_Metoprolol_ACTIVE	Filter	Knowledge	Boolean	Does the patient have an active Metoprolol beta blocker prescription on the index date?	0.0867
67	GUIDELINES_is_Beta_Blockers_Nadolol_ACTIVE	Filter	Knowledge	Boolean	Does the patient have an active Nadolol beta blocker prescription on the index date?	0.4588
68	GUIDELINES_is_Beta_Blockers_Nebivolol_ACTIVE	Filter	Knowledge	Boolean	Does the patient have an active Nebivolol beta blocker prescription on the index date?	0.0337
69	GUIDELINES_is_Beta_Blockers_Oxprenolol_ACTIVE	Filter	Knowledge	Boolean	Does the patient have an active Oxprenolol beta blocker prescription on the index date?	0.1170
70	GUIDELINES_is_Beta_Blockers_Penbutolol_ACTIVE	Filter	Knowledge	Boolean	Does the patient have an active Penbutolol beta blocker prescription on the index date?	0.8430
71	GUIDELINES_is_Beta_Blockers_Pindolol_ACTIVE	Filter	Knowledge	Boolean	Does the patient have an active Pindolol beta blocker prescription on the index date?	0.8505
72	GUIDELINES_is_Beta_Blockers_Propranolol_ACTIVE	Filter	Knowledge	Boolean	Does the patient have an active Propranolol beta blocker prescription on the index date?	0.1307
73	GUIDELINES_is_CCB_Amlodipine_ACTIVE	Filter	Knowledge	Boolean	Does the patient have an active Amlodipine calcium channel blocker prescription on the index date?	0.1575
74	GUIDELINES_is_CCB_Clevidipine_ACTIVE	Filter	Knowledge	Boolean	Does the patient have an active Clevidipine calcium channel blocker prescription on the index date?	1.2226
75	GUIDELINES_is_CCB_Diltiazem_ACTIVE	Filter	Knowledge	Boolean	Does the patient have an active Diltiazem calcium channel blocker prescription on the index date?	0.2289
76	GUIDELINES_is_CCB_Felodipine_ACTIVE	Filter	Knowledge	Boolean	Does the patient have an active Felodipine calcium channel blocker prescription on the index date?	0.0614
77	GUIDELINES_is_CCB_Isradipine_ACTIVE	Filter	Knowledge	Boolean	Does the patient have an active Isradipine calcium channel blocker prescription on the index date?	0.8797
78	GUIDELINES_is_CCB_Nicardipine_ACTIVE	Filter	Knowledge	Boolean	Does the patient have an active Nicardipine calcium channel blocker prescription on the index date?	0.5628
79	GUIDELINES_is_CCB_Nifedipine_ACTIVE	Filter	Knowledge	Boolean	Does the patient have an active Nifedipine calcium channel blocker prescription on the index date?	0.7946
80	GUIDELINES_is_CCB_Nimodipine_ACTIVE	Filter	Knowledge	Boolean	Does the patient have an active Nimodipine calcium channel blocker prescription on the index date?	1.0814
81	GUIDELINES_is_CCB_Nisoldipine_ACTIVE	Filter	Knowledge	Boolean	Does the patient have an active Nisoldipine calcium channel blocker prescription on the index date?	0.8639
82	GUIDELINES_is_CCB_Verapamil_ACTIVE	Filter	Knowledge	Boolean	Does the patient have an active Verapamil calcium channel blocker prescription on the index date?	0.0282
83	GUIDELINES_is_Central_Acting_Agents_Clonidine_ACTIVE	Filter	Knowledge	Boolean	Does the patient have an active Clonidine central acting prescription on the index date?	0.1535
84	GUIDELINES_is_Central_Acting_Agents_Deserpidine_ACTIVE	Filter	Knowledge	Boolean	Does the patient have an active Deserpidine central acting prescription on the index date?	1.1738
85	GUIDELINES_is_Central_Acting_Agents_Guanabenz_ACTIVE	Filter	Knowledge	Boolean	Does the patient have an active Guanabenz central acting prescription on the index date?	0.1264
86	GUIDELINES_is_Central_Acting_Agents_Guanfacine_ACTIVE	Filter	Knowledge	Boolean	Does the patient have an active Guanfacine central acting prescription on the index date?	0.6858
87	GUIDELINES_is_Central_Acting_Agents_Methyldopa_ACTIVE	Filter	Knowledge	Boolean	Does the patient have an active Methyldopa central acting prescription on the index date?	0.6347
88	GUIDELINES_is_Central_Acting_Agents_Reserpine_ACTIVE	Filter	Knowledge	Boolean	Does the patient have an active Reserpine central acting prescription on the index date?	0.5611
89	GUIDELINES_is_K_Sparing_Diuretics_Amiloride_ACTIVE	Filter	Knowledge	Boolean	Does the patient have an active Amiloride potassium sparing diuretic prescription on the index date?	0.4679
90	GUIDELINES_is_K_Sparing_Diuretics_Eplerenone_ACTIVE	Filter	Knowledge	Boolean	Does the patient have an active Eplerenone potassium sparing diuretic prescription on the index date?	0.4679
91	GUIDELINES_is_K_Sparing_Diuretics_Spironolactone_ACTIVE	Filter	Knowledge	Boolean	Does the patient have an active Spironolactone potassium sparing diuretic prescription on the index date?	1.0666
92	GUIDELINES_is_K_Sparing_Diuretics_Triamterene_ACTIVE	Filter	Knowledge	Boolean	Does the patient have an active Triamterene potassium sparing diuretic prescription on the index date?	0.1903
93	GUIDELINES_is_Loop_Diuretics_Bumetanide_ACTIVE	Filter	Knowledge	Boolean	Does the patient have an active Bumetanide loop diuretic prescription on the index date?	0.6982
94	GUIDELINES_is_Loop_Diuretics_Ethacrynate_ACTIVE	Filter	Knowledge	Boolean	Does the patient have an active Ethacrynate loop diuretic prescription on the index date?	0.6056
95	GUIDELINES_is_Loop_Diuretics_Furosemide_ACTIVE	Filter	Knowledge	Boolean	Does the patient have an active Furosemide loop diuretic prescription on the index date?	0.5258
96	GUIDELINES_is_Loop_Diuretics_Torsemide_ACTIVE	Filter	Knowledge	Boolean	Does the patient have an active Torsemide loop diuretic prescription on the index date?	1.1999
97	GUIDELINES_is_Neprilysin_Inhibitors_Sacubitril_ACTIVE	Filter	Knowledge	Boolean	Does the patient have an active Sacubitril Neprilysin Inhibitor prescription on the index date?	0.2792
98	GUIDELINES_is_Renin_inhibitors_Aliskiren_ACTIVE	Filter	Knowledge	Boolean	Does the patient have an active Aliskiren Renin Inhibitor prescription on the index date?	0.0078
99	GUIDELINES_is_Thiazide_Bendroflumethiazide_ACTIVE	Filter	Knowledge	Boolean	Does the patient have an active Bendroflumethiazide thiazide diuretic prescription on the index date?	0.9577
100	GUIDELINES_is_Thiazide_Chlorothiazide_ACTIVE	Filter	Knowledge	Boolean	Does the patient have an active Chlorothiazide thiazide diuretic prescription on the index date?	0.0810
101	GUIDELINES_is_Thiazide_Chlorthalidone_ACTIVE	Filter	Knowledge	Boolean	Does the patient have an active Chlorthalidone thiazide diuretic prescription on the index date?	0.2043
102	GUIDELINES_is_Thiazide_Hydrochlorothiazide_ACTIVE	Filter	Knowledge	Boolean	Does the patient have an active Hydrochlorothiazide thiazide diuretic prescription on the index date?	1.0610
103	GUIDELINES_is_Thiazide_Hydroflumethiazide_ACTIVE	Filter	Knowledge	Boolean	Does the patient have an active Hydroflumethiazide thiazide diuretic prescription on the index date?	0.0346
104	GUIDELINES_is_Thiazide_Indapamide_ACTIVE	Filter	Knowledge	Boolean	Does the patient have an active Indapamide thiazide diuretic prescription on the index date?	0.3921
105	GUIDELINES_is_Thiazide_Methyclothiazide_ACTIVE	Filter	Knowledge	Boolean	Does the patient have an active Methyclothiazide thiazide diuretic prescription on the index date?	0.5460
106	GUIDELINES_is_Thiazide_Metolazone_ACTIVE	Filter	Knowledge	Boolean	Does the patient have an active Metolazone thiazide diuretic prescription on the index date?	0.1264
107	GUIDELINES_is_Thiazide_Polythiazide_ACTIVE	Filter	Knowledge	Boolean	Does the patient have an active Polythiazide thiazide diuretic prescription on the index date?	0.7933
108	GUIDELINES_is_Thiazide_Trichlormethiazide_ACTIVE	Filter	Knowledge	Boolean	Does the patient have an active Trichlormethiazide thiazide diuretic prescription on the index date?	0.2699
109	GUIDELINES_is_Vasodilators_Fenoldopam_ACTIVE	Filter	Knowledge	Boolean	Does the patient have an active Fenoldopam vassodialator prescription on the index date?	0.7595
110	GUIDELINES_is_Vasodilators_Guanadrel_ACTIVE	Filter	Knowledge	Boolean	Does the patient have an active Guanadrel vassodialator prescription on the index date?	0.1751
111	GUIDELINES_is_Vasodilators_Hydralazine_ACTIVE	Filter	Knowledge	Boolean	Does the patient have an active Hydralazine vassodialator prescription on the index date?	0.3297
112	GUIDELINES_is_Vasodilators_Isosorbide_dinitrate_ACTIVE	Filter	Knowledge	Boolean	Does the patient have an active Isosorbide_dinitrate vassodialator prescription on the index date?	0.3594
113	GUIDELINES_is_Vasodilators_Minoxidil_ACTIVE	Filter	Knowledge	Boolean	Does the patient have an active Minoxidil vassodialator prescription on the index date?	0.4228
114	GUIDELINES_is_Vasodilators_Nitroprusside_ACTIVE	Filter	Knowledge	Boolean	Does the patient have an active Nitroprusside vassodialator prescription on the index date?	0.2805

Supplementary Table S1: The selected variables for hypertension (HTN). For each variable, the use (filtering or similarity), source (knowledge or data), type (Boolean or continuous), description, and similarity weights are provided.

N	Variable Name	Use	Source	Type	Description	Similarity Weight
1	DATA_count_14mo_A1C	Similarity	Data	Continuous	Number of hemoglobin A1C (HbA1c) lab tests in the past 14 months (from index date)	0.0884
2	DATA_count_14mo_cbc_panel	Similarity	Data	Continuous	Number of complete blood count (CBC) panel lab tests in the past 14 months (from index date)	0.0038
3	DATA_count_14mo_INR	Similarity	Data	Continuous	Number of prothrombin time international normalized ratio (INR) lab tests in the past 14 months (from index date)	0.0132
4	DATA_count_14mo_Preceding_Office_Visits_or_TELEPHONE_or_EMAIL	Similarity	Data	Continuous	Number of office visit, telephone, or email encounters in the past 14 months (from index date)	0.0397
5	DATA_count_antidiabetic_ACTIVE_meds	Similarity	Data	Continuous	Number of active antidiabetic medication prescriptions on the index date	0.2396
6	DATA_is_insurance_PRIVATE	Similarity	Data	Boolean	Does the patient have private health insurance on the index date?	0.9991
7	DATA_is_most_recent_Glucose_High	Similarity	Data	Boolean	Is the most recent (within 12 months) blood glucose lab test result high?	1.0011
8	DATA_is_most_recent_HDL_Cholesterol_High	Similarity	Data	Boolean	Is the most recent (within 12 months) high density lipid (HDL) Cholesterol test high?	0.9971
9	DATA_is_most_recent_Sodium_Low	Similarity	Data	Boolean	Is the most recent (within 12 months) sodium lab test low?	1.0007
10	DATA_most_recent_A1C	Similarity	Data	Continuous	The most recent (within 12 months) hemoglobin A1C (HbA1c) lab test result	0.1385
11	KNOWLEDGE_is_age_60_or_above_AND_is_PROC_Colonoscopy	Similarity	Knowledge	Boolean	Is patient age >=60 on the index date and completed a colonoscopy procedure within the past 11 years?	1.0029
12	KNOWLEDGE_is_hyperlipidemia_AND_is_most_recent_LDL_Cholesterol_NormalorLow	Similarity	Knowledge	Boolean	Is the patient diagnosed with hyperlipidemia before the index date and is the most recent (within 12 months) low density lipid (LDL) test normal or low?	0.9954
13	GUIDELINES__is_BMI_below_25	Filter	Knowledge	Boolean	Is the most recent (within 12 months) body mass index (BMI) below 25?	0.9955
14	GUIDELINES__is_Diabetic_Nephropathy	Filter	Knowledge	Boolean	Is the patient diagnosed with Diabetic Nephropathy before the index date?	0.9970
15	GUIDELINES__is_Diabetic_Neuropathy	Filter	Knowledge	Boolean	Is the patient diagnosed with Diabetic Neuropathy before the index date?	1.0002
16	GUIDELINES__is_Diabetic_Retinopathy	Filter	Knowledge	Boolean	Is the patient diagnosed with Diabetic Retinopathy before the index date?	0.9965
17	GUIDELINES__is_eGFR_below_30	Filter	Knowledge	Boolean	Is the most recent (within 12 months) Estimated Glomerular Filtration Rate (eGFR) below 30?	0.9962
18	GUIDELINES_is_Alpha_Glucosidase_Inhibitors_Acarbose_ACTIVE	Filter	Knowledge	Boolean	Does the patient have an active Acarbose Alpha Glucosidase Inhibitor prescription on the index date?	0.9647
19	GUIDELINES_is_Alpha_Glucosidase_Inhibitors_Miglitol_ACTIVE	Filter	Knowledge	Boolean	Does the patient have an active Miglitol Alpha Glucosidase Inhibitor prescription on the index date?	1.0019
20	GUIDELINES_is_Amylin_Analogs_Pramlintide_ACTIVE	Filter	Knowledge	Boolean	Does the patient have an active Pramlintide Amylin Analog prescription on the index date?	0.9206
21	GUIDELINES_is_Biguanides_Metformin_ACTIVE	Filter	Knowledge	Boolean	Does the patient have an active Metformin Biguanides prescription on the index date?	0.9696
22	GUIDELINES_is_Bile_acid_Sequestrants_Colesevelam_ACTIVE	Filter	Knowledge	Boolean	Does the patient have an active Colesevelam Bile Acid Sequestrant prescription on the index date?	0.9512
23	GUIDELINES_is_Dopamine_Receptor_Agonist_Bromocriptine_ACTIVE	Filter	Knowledge	Boolean	Does the patient have an active Bromocriptine Dopamine Receptor Agonist prescription on the index date?	0.5748
24	GUIDELINES_is_DPP_4_inhibitors_Alogliptin_ACTIVE	Filter	Knowledge	Boolean	Does the patient have an active Alogliptin dipeptidyl peptidase 4 (DPP4) prescription on the index date?	0.0499
25	GUIDELINES_is_DPP_4_inhibitors_Linagliptin_ACTIVE	Filter	Knowledge	Boolean	Does the patient have an active Linagliptin dipeptidyl peptidase 4 (DPP4) prescription on the index date?	0.9744
26	GUIDELINES_is_DPP_4_inhibitors_Saxagliptin_ACTIVE	Filter	Knowledge	Boolean	Does the patient have an active Saxagliptin dipeptidyl peptidase 4 (DPP4) prescription on the index date?	0.0224
27	GUIDELINES_is_DPP_4_inhibitors_Sitagliptin_ACTIVE	Filter	Knowledge	Boolean	Does the patient have an active Sitagliptin dipeptidyl peptidase 4 (DPP4) prescription on the index date?	0.9811
28	GUIDELINES_is_GLP_1_agonists_Albiglutide_ACTIVE	Filter	Knowledge	Boolean	Does the patient have an active Albiglutide Glucagon-like peptide-1 receptor agonists (GLP1) prescription on the index date?	0.0096
29	GUIDELINES_is_GLP_1_agonists_Dulaglutide_ACTIVE	Filter	Knowledge	Boolean	Does the patient have an active Dulaglutide Glucagon-like peptide-1 receptor agonists (GLP1) prescription on the index date?	0.9492
30	GUIDELINES_is_GLP_1_agonists_Exenatide_ACTIVE	Filter	Knowledge	Boolean	Does the patient have an active Exenatide Glucagon-like peptide-1 receptor agonists (GLP1) prescription on the index date?	0.9649
31	GUIDELINES_is_GLP_1_agonists_Liraglutide_ACTIVE	Filter	Knowledge	Boolean	Does the patient have an active Liraglutide Glucagon-like peptide-1 receptor agonists (GLP1) prescription on the index date?	0.9808
32	GUIDELINES_is_GLP_1_agonists_Lixisenatide_ACTIVE	Filter	Knowledge	Boolean	Does the patient have an active Lixisenatide Glucagon-like peptide-1 receptor agonists (GLP1) prescription on the index date?	0.0421
33	GUIDELINES_is_GLP_1_agonists_Semaglutide_ACTIVE	Filter	Knowledge	Boolean	Does the patient have an active Semaglutide Glucagon-like peptide-1 receptor agonists (GLP1) prescription on the index date?	0.3186
34	GUIDELINES_is_Insulin_Aspart_Human_ACTIVE	Filter	Knowledge	Boolean	Does the patient have an active Aspart Human insulin prescription on the index date?	0.8088
35	GUIDELINES_is_Insulin_Aspart_Protamine_Human_ACTIVE	Filter	Knowledge	Boolean	Does the patient have an active Aspart Protamine Human insulin prescription on the index date?	0.1768
36	GUIDELINES_is_Insulin_Degludec_ACTIVE	Filter	Knowledge	Boolean	Does the patient have an active Degludec insulin prescription on the index date?	1.2390
37	GUIDELINES_is_Insulin_Detemir_ACTIVE	Filter	Knowledge	Boolean	Does the patient have an active Detemir insulin prescription on the index date?	0.9526
38	GUIDELINES_is_Insulin_Glargine_ACTIVE	Filter	Knowledge	Boolean	Does the patient have an active Glargine insulin prescription on the index date?	0.9742
39	GUIDELINES_is_Insulin_Glulisine_Human_ACTIVE	Filter	Knowledge	Boolean	Does the patient have an active Glulisine Human insulin prescription on the index date?	1.1951
40	GUIDELINES_is_Insulin_Human_rDNA_origin_ACTIVE	Filter	Knowledge	Boolean	Does the patient have an active Human rDNA origin insulin prescription on the index date?	0.9567
41	GUIDELINES_is_Insulin_Isophane_ACTIVE	Filter	Knowledge	Boolean	Does the patient have an active Isophane insulin prescription on the index date?	0.1574
42	GUIDELINES_is_Insulin_Lispro_ACTIVE	Filter	Knowledge	Boolean	Does the patient have an active Lispro insulin prescription on the index date?	0.9847
43	GUIDELINES_is_Insulin_Prompt_Zinc_Human_ACTIVE	Filter	Knowledge	Boolean	Does the patient have an active Prompt Zinc Human insulin prescription on the index date?	1.2867
44	GUIDELINES_is_Insulin_Protamine_Lispro_Human_ACTIVE	Filter	Knowledge	Boolean	Does the patient have an active Protamine_Lispro_Human insulin prescription on the index date?	0.9854
45	GUIDELINES_is_Insulin_Protamine_Zinc_Human_ACTIVE	Filter	Knowledge	Boolean	Does the patient have an active Protamine Zinc Human insulin prescription on the index date?	1.0945
46	GUIDELINES_is_Insulin_Regular_Human_ACTIVE	Filter	Knowledge	Boolean	Does the patient have an active Regular Human insulin prescription on the index date?	0.9301
47	GUIDELINES_is_Meglitinides_Nateglinide_ACTIVE	Filter	Knowledge	Boolean	Does the patient have an active Nateglinide Meglitinide prescription on the index date?	0.9560
48	GUIDELINES_is_Meglitinides_Repaglinide_ACTIVE	Filter	Knowledge	Boolean	Does the patient have an active Repaglinide Meglitinide prescription on the index date?	0.9758
49	GUIDELINES_is_SGLT2_Inhibitors_Canagliflozin_ACTIVE	Filter	Knowledge	Boolean	Does the patient have an active Canagliflozin Sodium-glucose Cotransporter-2 (SGLT2) prescription on the index date?	0.9307
50	GUIDELINES_is_SGLT2_Inhibitors_Dapagliflozin_ACTIVE	Filter	Knowledge	Boolean	Does the patient have an active Dapagliflozin Sodium-glucose Cotransporter-2 (SGLT2) prescription on the index date?	0.4349
51	GUIDELINES_is_SGLT2_Inhibitors_Empagliflozin_ACTIVE	Filter	Knowledge	Boolean	Does the patient have an active Empagliflozin Sodium-glucose Cotransporter-2 (SGLT2) prescription on the index date?	0.9689
52	GUIDELINES_is_SGLT2_Inhibitors_Ertugliflozin_ACTIVE	Filter	Knowledge	Boolean	Does the patient have an active Ertugliflozin Sodium-glucose Cotransporter-2 (SGLT2) prescription on the index date?	0.9948
53	GUIDELINES_is_Sulfonylureas_Chlorpropamide_ACTIVE	Filter	Knowledge	Boolean	Does the patient have an active Chlorpropamide Sulfonylurea prescription on the index date?	0.1407
54	GUIDELINES_is_Sulfonylureas_Glimepiride_ACTIVE	Filter	Knowledge	Boolean	Does the patient have an active Glimepiride Sulfonylurea prescription on the index date?	0.9694
55	GUIDELINES_is_Sulfonylureas_Glipizide_ACTIVE	Filter	Knowledge	Boolean	Does the patient have an active Glipizide Sulfonylurea prescription on the index date?	0.9647
56	GUIDELINES_is_Sulfonylureas_Glyburide_ACTIVE	Filter	Knowledge	Boolean	Does the patient have an active Glyburide Sulfonylurea prescription on the index date?	0.9679
57	GUIDELINES_is_Sulfonylureas_Tolazamide_ACTIVE	Filter	Knowledge	Boolean	Does the patient have an active Tolazamide Sulfonylurea prescription on the index date?	0.1798
58	GUIDELINES_is_Sulfonylureas_Tolbutamide_ACTIVE	Filter	Knowledge	Boolean	Does the patient have an active Tolbutamide Sulfonylurea prescription on the index date?	0.3657
59	GUIDELINES_is_TZDs_Pioglitazone_ACTIVE	Filter	Knowledge	Boolean	Does the patient have an active Pioglitazone Thiazolidinediones (TZD) prescription on the index date?	0.9694
60	GUIDELINES_is_TZDs_Rosiglitazone_ACTIVE	Filter	Knowledge	Boolean	Does the patient have an active Rosiglitazone Thiazolidinediones (TZD) prescription on the index date?	0.9621

Supplementary Table S2: The selected variables for type 2 diabetes mellitus (T2DM). For each variable, the use (filtering or similarity), source (knowledge or data), type (Boolean or continuous), description, and similarity weights are provided.

N	Variable Name	Use	Source	Type	Description	Similarity Weight
1	DATA_count_14mo_A1C	Similarity	Data	Continuous	Number of hemoglobin A1C (HbA1c) lab tests in the past 14 months (from index date)	0.0318
2	DATA_count_14mo_INR	Similarity	Data	Continuous	Number of prothrombin time international normalized ratio (INR) lab tests in the past 14 months (from index date)	0.4816
3	DATA_count_14mo_LDL_Cholesterol	Similarity	Data	Continuous	Number of low density lipid (LDL) Cholesterol lab tests in the past 14 months (from index date)	0.3332
4	DATA_count_14mo_Potassium	Similarity	Data	Continuous	Number of potassium lab tests in the past 14 months (from index date)	0.0988
5	DATA_count_14mo_Preceding_Admissions	Similarity	Data	Continuous	Number of hospital admissions in the past 14 months (from index date)	0.2191
6	DATA_count_14mo_Preceding_ER_Visits	Similarity	Data	Continuous	Number of emergency room/department encounters in the past 14 months (from index date)	0.0800
7	DATA_count_antihyperlipidemic_ACTIVE_meds	Similarity	Data	Continuous	Number of active antihyperlipidemic medication prescriptions on the index date	0.2569
8	DATA_is_age_60_or_above	Similarity	Data	Boolean	Is the patient age >= 60 on the index date?	0.4055
9	DATA_is_Dysrhythmia	Similarity	Data	Boolean	Is the patient diagnosed with Dysrhythmia before the index date?	1.0170
10	DATA_is_History_Admissions	Similarity	Data	Boolean	Does patient have a prior hospital admission encounter (from index date)?	0.8995
11	DATA_is_HTN	Similarity	Data	Boolean	Is the patient diagnosed with hypertension (HTN) before the index date?	0.1885
12	DATA_is_insurance_PRIVATE	Similarity	Data	Boolean	Does the patient have private health insurance on the index date?	0.4775
13	DATA_is_male	Similarity	Data	Boolean	Is the patient sex male?	0.2589
14	DATA_is_most_recent_A1C_High	Similarity	Data	Boolean	Is the most recent (within 12 months) hemoglobin A1C (HbA1c) test high?	0.0734
15	DATA_is_most_recent_BMI_Normal	Similarity	Data	Boolean	Is the most recent (within 12 months) body mass index (BMI) normal?	0.0671
16	DATA_is_most_recent_eGFR_Low	Similarity	Data	Boolean	Is the most recent (within 12 months) Estimated Glomerular Filtration Rate (eGFR) low?	0.6944
17	DATA_is_most_recent_HDL_Cholesterol_Low	Similarity	Data	Boolean	Is the most recent (within 12 months) high density lipid (HDL) Cholesterol test low?	0.1922
18	DATA_is_most_recent_Hemoglobin_Normal	Similarity	Data	Boolean	Is the most recent (within 12 months) Hemoglobin lab test normal?	0.1712
19	DATA_is_most_recent_LDL_Cholesterol_High	Similarity	Data	Boolean	Is the most recent (within 12 months) low density lipid (LDL) Cholesterol test high?	0.6286
20	DATA_is_most_recent_Potassium_Normal	Similarity	Data	Boolean	Is the most recent (within 12 months) potassium lab test normal?	0.0548
21	DATA_is_most_recent_Triglyceride_High	Similarity	Data	Boolean	Is the most recent (within 12 months) triglyceride test high?	0.0472
22	DATA_PROC_is_Immunization	Similarity	Data	Boolean	Did the patient have an immunization procedure in the past 12 months (from index date)	0.2291
23	GUIDELINES__is_ASCVD	Filter	Knowledge	Boolean	Is the patient diagnosed with Atherosclerotic Cardiovascular Disease (ASCVD) before the index date?	0.9204
24	GUIDELINES__is_T2D	Filter	Knowledge	Boolean	Is the patient diagnosed with type 2 diabetes (T2D) before the index date?	0.2864
25	GUIDELINES_is_Antilipemic_Agent_Misc_ACTIVE	Filter	Knowledge	Boolean	Does patient have an active Miscellaneous Antilipemic Agent prescription on index date?	0.0001
26	GUIDELINES_is_Bile_Acid_Sequestrant_ACTIVE	Filter	Knowledge	Boolean	Does patient have an active Bile Acid Sequestrant prescription on index date?	0.3094
27	GUIDELINES_is_Cholesterol_Absorption_Inhibitor_ACTIVE	Filter	Knowledge	Boolean	Does patient have an active Cholesterol Absorption Inhibitor prescription on index date?	0.8460
28	GUIDELINES_is_Fibric_Acid_Derivative_ACTIVE	Filter	Knowledge	Boolean	Does patient have an active Fibric Acid Derivative prescription on index date?	0.4595
29	GUIDELINES_is_PCSK9_ACTIVE	Filter	Knowledge	Boolean	Does patient have an active Proprotein convertase subtilisin/kexin type 9 (PCSK9) prescription on index date?	0.9830
30	GUIDELINES_is_Statin_Atorvastatin_ACTIVE	Filter	Knowledge	Boolean	Does patient have an active Atorvastatin statin prescription on index date?	1.3313
31	GUIDELINES_is_Statin_Fluvastatin_ACTIVE	Filter	Knowledge	Boolean	Does patient have an active Fluvastatin statin prescription on index date?	0.9029
32	GUIDELINES_is_Statin_Lovastatin_ACTIVE	Filter	Knowledge	Boolean	Does patient have an active Lovastatin statin prescription on index date?	1.1416
33	GUIDELINES_is_Statin_Pitavastatin_ACTIVE	Filter	Knowledge	Boolean	Does patient have an active Pitavastatin statin prescription on index date?	0.6500
34	GUIDELINES_is_Statin_Pravastatin_ACTIVE	Filter	Knowledge	Boolean	Does patient have an active Pravastatin statin prescription on index date?	0.5837
35	GUIDELINES_is_Statin_Rosuvastatin_ACTIVE	Filter	Knowledge	Boolean	Does patient have an active Rosuvastatin statin prescription on index date?	1.0377
36	GUIDELINES_is_Statin_Simvastatin_ACTIVE	Filter	Knowledge	Boolean	Does patient have an active Simvastatin statin prescription on index date?	0.9841

Supplementary Table S3: The selected variables for hyperlipidemia (HL). For each variable, the use (filtering or similarity), source (knowledge or data), type (Boolean or continuous), description, and similarity weights are provided.

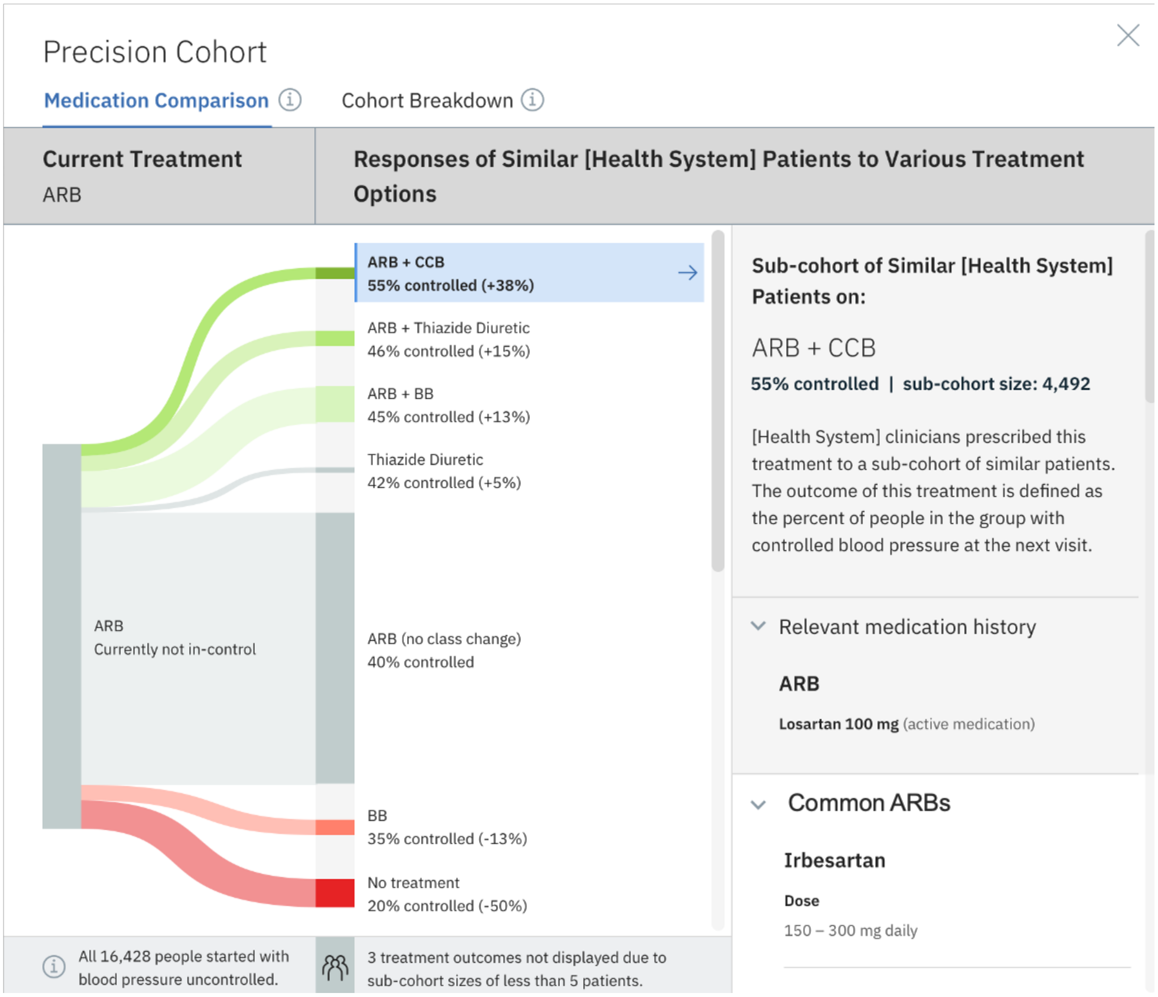

Supplementary Figure S4: Precision cohort visualization in the EHR. The large gray cohort in the middle represents decision points where no change was made to the treatment drug class. Green arms show alternative medication class treatment decisions in similar patients which led to better clinical outcomes. Red arms show alternatives that led to worse clinical outcomes. The dark green and dark red arms were statistically significant.

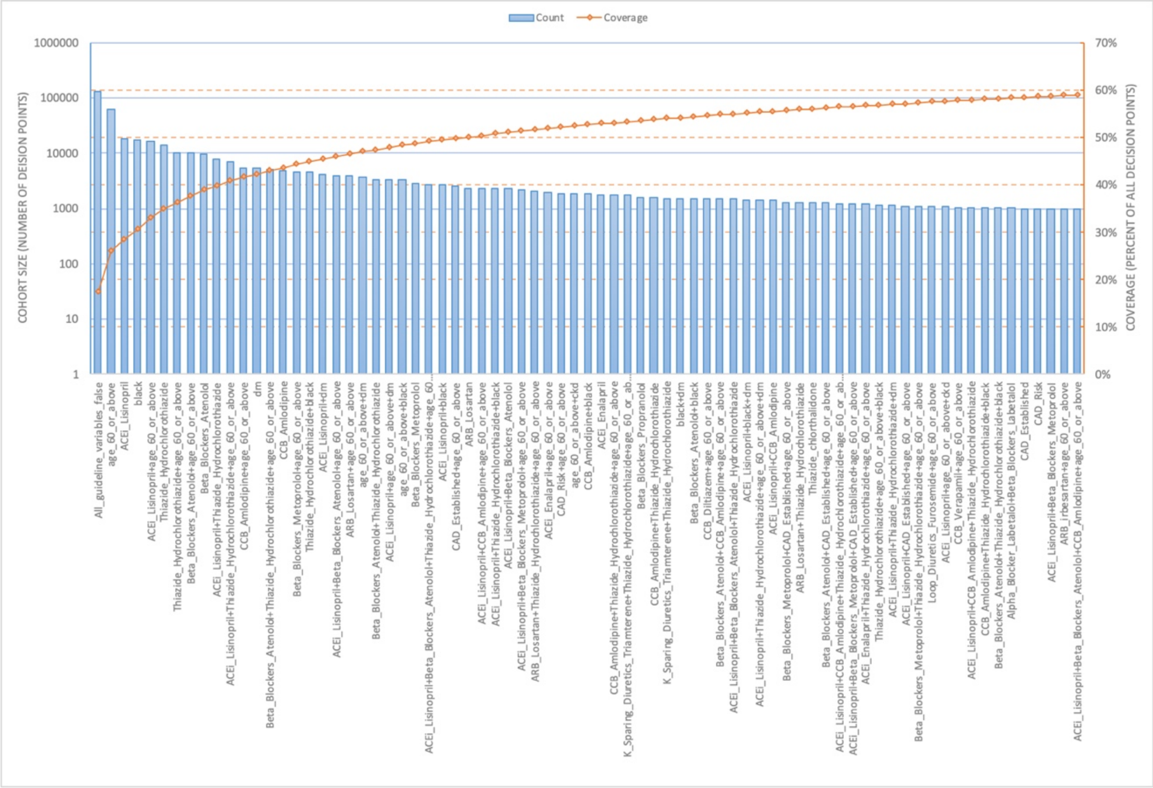

Supplementary Figure S5: The 75 largest hypertension (HTN) cohorts based on filter variables. The vertical blue bars show the cohort size (number of decision points) on a log scale (left vertical axis). The orange line graph shows the cumulative coverage on a linear percentage scale (right vertical axis).

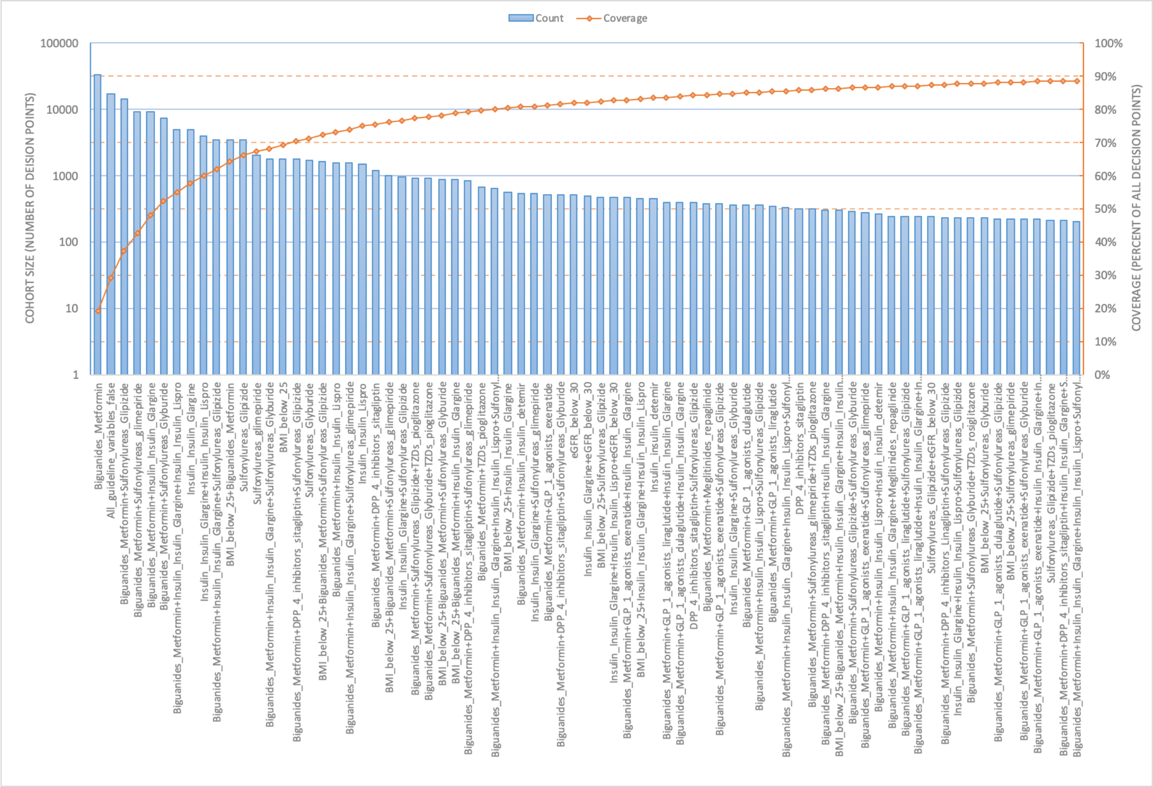

Supplementary Figure S6: The 75 largest type 2 diabetes mellitus (T2DM) cohorts based on filter variables. The vertical blue bars show the cohort size (number of decision points) on a log scale (left vertical axis). The orange line graph shows the cumulative coverage on a linear percentage scale (right vertical axis).

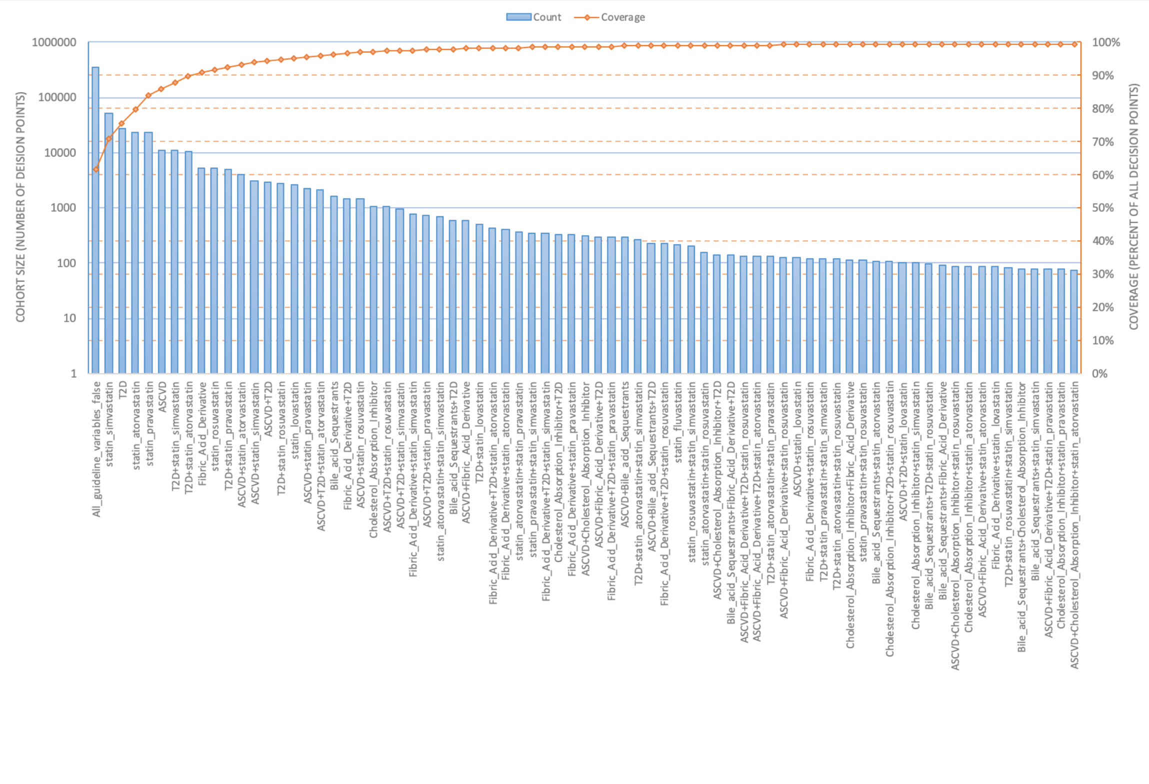

Supplementary Figure S7: The 75 largest hyperlipidemia (HL) cohorts based on filter variables. The vertical blue bars show the cohort size (number of decision points) on a log scale (left vertical axis). The orange line graph shows the cumulative coverage on a linear percentage scale (right vertical axis).

N	Treatment Option Group	Count	Percentage	Percent Controlled
0	No Change	606487	81.21%	46.91%
1	ACEi_Lisinopril_NEW	24247	3.25%	57.28%
2	Thiazide_Hydrochlorothiazide_NEW	20044	2.68%	52.29%
3	CCB_Amlodipine_NEW	14612	1.96%	51.93%
4	Beta_Blockers_Atenolol_NEW	6135	0.82%	48.04%
5	ACEi_Lisinopril_STOP	4261	0.57%	42.38%
6	ARB_Losartan_NEW	3952	0.53%	50.38%
7	Beta_Blockers_Metoprolol_NEW	3897	0.52%	45.21%
8	Thiazide_Hydrochlorothiazide_STOP	3845	0.51%	40.70%
9	Thiazide_chlorthalidone_NEW	3031	0.41%	57.74%
10	CCB_Amlodipine_STOP	2522	0.34%	39.49%
11	ACEi_Lisinopril_NEW+Thiazide_Hydrochlorothiazide_NEW	2248	0.30%	62.37%
12	Beta_Blockers_Atenolol_STOP	2108	0.28%	41.75%
13	Loop_Diuretics_Furosemide_NEW	2056	0.28%	45.38%
14	ACEi_Enalapril_NEW	1551	0.21%	47.39%
15	Beta_Blockers_Metoprolol_STOP	1397	0.19%	42.88%
16	ARB_irbesartan_NEW	1335	0.18%	43.60%
17	Loop_Diuretics_Furosemide_STOP	1136	0.15%	43.05%
18	ARB_Losartan_STOP	1079	0.14%	39.20%
19	Alpha_Blocker_Labetalol_NEW+Beta_Blockers_Labetalol_NEW	1077	0.14%	39.46%
20	K_Sparing_Diuretics_Triamterene_NEW+Thiazide_Hydrochlorothiazide_NEW	1054	0.14%	51.33%
21	CCB_Nifedipine_NEW	1008	0.13%	47.12%
22	Aldosterone_Receptor_Blocker_Spironolactone_NEW+K_Sparing_Diuretics_Spironolactone_NEW	966	0.13%	48.24%
23	CCB_Diltiazem_NEW	965	0.13%	40.21%
24	Central_Acting_Agents_Clonidine_NEW	706	0.09%	40.23%
25	ACEi_Lisinopril_STOP+ARB_Losartan_NEW	643	0.09%	48.06%
26	CCB_Verapamil_NEW	627	0.08%	40.83%
27	ACEi_Enalapril_STOP	596	0.08%	38.26%
28	Alpha_Blocker_Terazosin_NEW	589	0.08%	38.37%
29	ARB_valsartan_NEW	569	0.08%	40.95%
30	ACEi_Lisinopril_NEW+Thiazide_Hydrochlorothiazide_STOP	558	0.07%	53.94%
31	Alpha_Blocker_carvedilol_NEW+Beta_Blockers_carvedilol_NEW	555	0.07%	40.00%
32	Beta_Blockers_Propranolol_NEW	554	0.07%	53.61%
33	Vasodilators_Hydralazine_NEW	514	0.07%	33.46%
34	Thiazide_Hydrochlorothiazide_STOP+Thiazide_chlorthalidone_NEW	509	0.07%	46.95%
35	Thiazide_chlorthalidone_STOP	488	0.07%	40.57%
36	ACEi_Lisinopril_STOP+Thiazide_Hydrochlorothiazide_STOP	486	0.07%	45.27%
37	ACEi_Lisinopril_STOP+CCB_Amlodipine_NEW	480	0.06%	44.58%
38	ARB_irbesartan_STOP	446	0.06%	40.58%
39	Alpha_Blocker_Doxazosin_NEW	442	0.06%	38.91%
40	CCB_Diltiazem_STOP	430	0.06%	40.47%
41	K_Sparing_Diuretics_Triamterene_STOP+Thiazide_Hydrochlorothiazide_STOP	402	0.05%	39.30%
42	Loop_Diuretics_torsemide_NEW	383	0.05%	48.30%
43	ARB_Losartan_NEW+Thiazide_Hydrochlorothiazide_NEW	371	0.05%	57.95%
44	CCB_Amlodipine_NEW+Thiazide_Hydrochlorothiazide_STOP	368	0.05%	48.10%
45	Alpha_Blocker_Labetalol_STOP+Beta_Blockers_Labetalol_STOP	349	0.05%	38.68%
46	CCB_Nifedipine_STOP	338	0.05%	32.25%
47	ARB_valsartan_STOP	317	0.04%	39.43%
48	Central_Acting_Agents_Clonidine_STOP	303	0.04%	38.94%
49	Alpha_Blocker_Terazosin_STOP	299	0.04%	37.46%
50	CCB_Amlodipine_NEW+Thiazide_Hydrochlorothiazide_NEW	296	0.04%	50.68%

Supplementary Figure S8: The 50 largest observed global treatment option groups across all the decision points for hypertension (HTN). The size of each group (count and percentage of total) and the associated percent controlled during follow-up are shown. Treatment options with percent controlled higher than the "No Change" option are shaded in green while those with lower percent controlled are shaded in red.

N	Treatment Option Group	Count	Percent	PercentControlled
0	No Change	149184	84.98%	17.73%
1	Biguanides_Metformin_NEW	5377	3.06%	37.29%
2	Sulfonylureas_Glipizide_NEW	2087	1.19%	29.61%
3	Insulin_Insulin_Glargine_NEW	1920	1.09%	12.55%
4	Sulfonylureas_glimepiride_NEW	1838	1.05%	32.81%
5	Biguanides_Metformin_STOP	1470	0.84%	21.77%
6	Insulin_Insulin_Glargine_STOP	1017	0.58%	10.82%
7	DPP_4_inhibitors_sitagliptin_NEW	1004	0.57%	22.21%
8	Sulfonylureas_Glipizide_STOP	776	0.44%	17.27%
9	Insulin_Insulin_Lispro_NEW	713	0.41%	8.70%
10	Sulfonylureas_glimepiride_STOP	570	0.32%	15.61%
11	Sulfonylureas_Glyburide_STOP	515	0.29%	16.12%
12	Sulfonylureas_Glyburide_NEW	480	0.27%	25.42%
13	Insulin_Insulin_Lispro_STOP	410	0.23%	9.51%
14	GLP_1_agonists_dulaglutide_NEW	405	0.23%	24.44%
15	TZDs_pioglitazone_NEW	400	0.23%	26.00%
16	GLP_1_agonists_exenatide_NEW	356	0.20%	18.26%
17	DPP_4_inhibitors_sitagliptin_STOP	333	0.19%	10.81%
18	TZDs_pioglitazone_STOP	286	0.16%	10.84%
19	GLP_1_agonists_liraglutide_NEW	257	0.15%	19.84%
20	Sulfonylureas_Glipizide_NEW+Sulfonylureas_Glyburide_STOP	250	0.14%	13.20%
21	Biguanides_Metformin_NEW+Sulfonylureas_Glipizide_NEW	238	0.14%	30.67%
22	GLP_1_agonists_exenatide_STOP	200	0.11%	11.00%
23	SGLT2_Inhibitors_empagliflozin_NEW	198	0.11%	17.68%
24	SGLT2_Inhibitors_canagliflozin_NEW	161	0.09%	15.53%
25	DPP_4_inhibitors_Linagliptin_NEW	135	0.08%	19.26%
26	Insulin_insulin_detemir_NEW	134	0.08%	14.18%
27	Meglitinides_repaglinide_NEW	123	0.07%	17.07%
28	Sulfonylureas_Glyburide_STOP+Sulfonylureas_glimepiride_NEW	113	0.06%	11.50%
29	GLP_1_agonists_liraglutide_STOP	112	0.06%	8.93%
30	Biguanides_Metformin_NEW+Sulfonylureas_glimepiride_NEW	109	0.06%	33.03%
31	Biguanides_Metformin_NEW+Insulin_Insulin_Glargine_NEW	108	0.06%	32.41%
32	Insulin_Insulin_Glargine_NEW+Insulin_Insulin_Lispro_NEW	105	0.06%	13.33%
33	Insulin_insulin_detemir_STOP	91	0.05%	9.89%
34	Biguanides_Metformin_NEW+Sulfonylureas_Glyburide_NEW	81	0.05%	22.22%
35	Meglitinides_repaglinide_STOP	81	0.05%	16.05%
36	Sulfonylureas_Glipizide_STOP+Sulfonylureas_glimepiride_NEW	80	0.05%	12.50%
37	Insulin_Insulin_Glargine_STOP+Insulin_Insulin_Lispro_STOP	67	0.04%	5.97%
38	SGLT2_Inhibitors_canagliflozin_STOP	66	0.04%	7.58%
39	Biguanides_Metformin_STOP+Sulfonylureas_Glipizide_STOP	65	0.04%	23.08%
40	Biguanides_Metformin_STOP+Sulfonylureas_glimepiride_NEW	64	0.04%	35.94%
41	TZDs_rosiglitazone_STOP	62	0.04%	9.68%
42	GLP_1_agonists_dulaglutide_STOP	58	0.03%	3.45%
43	Insulin_Insulin_Glargine_NEW+Sulfonylureas_Glipizide_STOP	58	0.03%	10.34%
44	Biguanides_Metformin_STOP+Sulfonylureas_Glipizide_NEW	56	0.03%	35.71%
45	Alpha_Glucosidase_Inhibitors_Acarbose_NEW	54	0.03%	7.41%
46	DPP_4_inhibitors_Linagliptin_STOP	49	0.03%	6.12%
47	Biguanides_Metformin_STOP+Sulfonylureas_Glyburide_STOP	48	0.03%	20.83%
48	Insulin_insulin_degludec_NEW	47	0.03%	8.51%
49	Biguanides_Metformin_NEW+DPP_4_inhibitors_sitagliptin_NEW	46	0.03%	26.09%
50	Sulfonylureas_Glipizide_NEW+Sulfonylureas_glimepiride_STOP	46	0.03%	15.22%

Supplementary Figure S9: The 50 largest observed global treatment option groups across all the decision points for type 2 diabetes mellitus (T2DM). The size of each group (count and percentage of total) and the associated percent controlled during follow-up are shown. Treatment options with percent controlled higher than the “No Change” option are shaded in green while those with lower percent controlled are shaded in red.

N	Treatment Option Group	Count	Percentage	PercentControlled
0	NoChange	528354	94.02%	34.71%
1	statin_simvastatin_NEW	8679	1.54%	68.21%
2	statin_atorvastatin_NEW	6438	1.15%	75.61%
3	statin_pravastatin_NEW	3943	0.70%	54.32%
4	statin_simvastatin_STOP	3081	0.55%	33.72%
5	statin_pravastatin_STOP	1621	0.29%	31.96%
6	statin_atorvastatin_STOP	1589	0.28%	36.19%
7	statin_rosuvastatin_NEW	1101	0.20%	62.94%
8	statin_atorvastatin_NEW+statin_simvastatin_STOP	1022	0.18%	71.04%
9	statin_rosuvastatin_STOP	536	0.10%	33.40%
10	statin_atorvastatin_NEW+statin_pravastatin_STOP	485	0.09%	77.94%
11	Fibric_Acid_Derivative_NEW	470	0.08%	38.94%
12	statin_pravastatin_NEW+statin_simvastatin_STOP	418	0.07%	48.56%
13	Fibric_Acid_Derivative_STOP	405	0.07%	44.94%
14	Bile_acid_Sequestrants_NEW	297	0.05%	33.67%
15	Cholesterol_Absorption_Inhibitor_NEW	287	0.05%	44.95%
16	Bile_acid_Sequestrants_STOP	253	0.05%	27.67%
17	statin_rosuvastatin_NEW+statin_simvastatin_STOP	239	0.04%	65.27%
18	statin_lovastatin_NEW	238	0.04%	52.10%
19	Cholesterol_Absorption_Inhibitor_STOP	205	0.04%	31.22%
20	statin_atorvastatin_STOP+statin_rosuvastatin_NEW	192	0.03%	61.46%
21	statin_lovastatin_STOP	178	0.03%	28.09%
22	statin_atorvastatin_NEW+statin_rosuvastatin_STOP	150	0.03%	62.00%
23	statin_atorvastatin_STOP+statin_pravastatin_NEW	140	0.02%	45.00%
24	statin_pravastatin_STOP+statin_simvastatin_NEW	106	0.02%	65.09%
25	statin_pravastatin_STOP+statin_rosuvastatin_NEW	97	0.02%	67.01%
26	statin_atorvastatin_STOP+statin_simvastatin_NEW	95	0.02%	58.95%
27	Fibric_Acid_Derivative_STOP+statin_atorvastatin_NEW	55	0.01%	89.09%
28	Fibric_Acid_Derivative_STOP+statin_simvastatin_NEW	55	0.01%	76.36%
29	statin_pravastatin_NEW+statin_rosuvastatin_STOP	55	0.01%	45.45%
30	statin_rosuvastatin_STOP+statin_simvastatin_NEW	48	0.01%	45.83%
31	statin_atorvastatin_NEW+statin_lovastatin_STOP	40	0.01%	77.50%
32	statin_atorvastatin_STOP+statin_simvastatin_STOP	36	0.01%	30.56%
33	statin_pravastatin_STOP+statin_simvastatin_STOP	33	0.01%	18.18%
34	statin_lovastatin_NEW+statin_pravastatin_STOP	29	0.01%	58.62%
35	statin_lovastatin_NEW+statin_simvastatin_STOP	29	0.01%	31.03%
36	statin_fluvastatin_NEW	26	0.00%	42.31%
37	Fibric_Acid_Derivative_STOP+statin_pravastatin_NEW	23	0.00%	73.91%
38	statin_lovastatin_STOP+statin_simvastatin_NEW	22	0.00%	86.36%
39	Fibric_Acid_Derivative_NEW+statin_simvastatin_STOP	21	0.00%	9.52%
40	statin_atorvastatin_STOP+statin_pravastatin_STOP	20	0.00%	25.00%
41	PCSK9_NEW	20	0.00%	45.00%
42	Fibric_Acid_Derivative_STOP+statin_simvastatin_STOP	20	0.00%	60.00%
43	statin_fluvastatin_STOP	19	0.00%	10.53%
44	Fibric_Acid_Derivative_NEW+statin_simvastatin_NEW	18	0.00%	66.67%
45	statin_rosuvastatin_STOP+statin_simvastatin_STOP	18	0.00%	38.89%
46	Cholesterol_Absorption_Inhibitor_STOP+statin_pravastatin_NEW	18	0.00%	33.33%
47	statin_atorvastatin_NEW+statin_simvastatin_NEW	17	0.00%	58.82%
48	Fibric_Acid_Derivative_STOP+statin_rosuvastatin_NEW	17	0.00%	64.71%
49	Cholesterol_Absorption_Inhibitor_STOP+statin_atorvastatin_NEW	15	0.00%	60.00%
50	Cholesterol_Absorption_Inhibitor_STOP+statin_simvastatin_STOP	15	0.00%	60.00%

Supplementary Figure S10: The 50 largest observed global treatment option groups across all the decision points for hyperlipidemia (HL). The size of each group (count and percentage of total) and the associated percent controlled during follow-up are shown. Treatment options with percent controlled higher than the "No Change" option are shaded in green while those with lower percent controlled are shaded in red.

Filter Variable Cohort	numTotal	Significantly Better Treatment Option	Treatment Option with Fewer Medications	Treatment Option with Same Medication Count	Treatment Option with More Medications
All Guideline Variables False	130361	99.49%	0.00%	0.00%	100.00%
age_60_or_above	63048	99.53%	0.00%	0.00%	100.00%
ACEi_Lisinopril	18313	99.56%	6.25%	57.30%	100.00%
black	17491	70.18%	0.00%	0.00%	100.00%
ACEi_Lisinopril+age_60_or_above	16701	95.46%	40.52%	96.35%	99.42%
Thiazide_Hydrochlorothiazide	14101	100.00%	57.30%	99.01%	100.00%
Thiazide_Hydrochlorothiazide+age_60_or_above	10346	97.14%	6.05%	95.85%	100.00%
Beta_Blockers_Atenolol+age_60_or_above	9943	72.96%	7.23%	80.12%	99.45%
Beta_Blockers_Atenolol	9635	65.24%	48.80%	82.95%	99.51%
ACEi_Lisinopril+Thiazide_Hydrochlorothiazide	7885	9.45%	93.13%	91.82%	96.74%
ACEi_Lisinopril+Thiazide_Hydrochlorothiazide+age_60_or_above	7203	64.24%	97.74%	93.67%	99.56%
CCB_Amlodipine+age_60_or_above	5367	32.64%	14.27%	74.64%	97.28%
dm	5291	78.23%	0.00%	0.00%	99.45%
Beta_Blockers_Atenolol+Thiazide_Hydrochlorothiazide+age_60_or_above	5029	49.53%	91.57%	84.65%	98.21%
CCB_Amlodipine	4902	68.16%	5.43%	77.87%	99.41%
Beta_Blockers_Metoprolol+age_60_or_above	4654	67.08%	58.79%	43.23%	99.66%
Thiazide_Hydrochlorothiazide+black	4590	35.77%	4.73%	93.01%	97.82%
ACEi_Lisinopril+dm	4124	21.44%	29.92%	40.28%	97.24%
ACEi_Lisinopril+Beta_Blockers_Atenolol+age_60_or_above	3987	62.63%	93.18%	71.83%	97.34%
ARB_Losartan+age_60_or_above	3932	83.11%	4.40%	1.83%	97.28%
age_60_or_above+dm	3726	71.52%	0.00%	0.00%	98.55%
Beta_Blockers_Atenolol+Thiazide_Hydrochlorothiazide	3425	80.91%	93.08%	93.23%	100.00%
ACEi_Lisinopril+age_60_or_above+dm	3422	71.54%	84.07%	68.32%	99.33%
age_60_or_above+black	3411	76.22%	0.00%	0.00%	98.33%
Beta_Blockers_Metoprolol	2912	8.07%	0.93%	68.34%	97.91%
ACEi_Lisinopril+Beta_Blockers_Atenolol+Thiazide_Hydrochlorothiazide+age_60_or_above	2765	4.41%	83.44%	80.90%	96.38%
ACEi_Lisinopril+black	2705	75.16%	81.07%	52.75%	100.00%
CAD_Established+age_60_or_above	2524	14.78%	0.00%	0.00%	88.47%
ARB_Losartan	2360	62.37%	1.02%	28.22%	99.15%
ACEi_Lisinopril+CCB_Amlodipine+age_60_or_above	2357	5.98%	95.84%	60.84%	88.93%
ACEi_Lisinopril+Thiazide_Hydrochlorothiazide+black	2277	8.70%	86.21%	33.29%	85.42%
ACEi_Lisinopril+Beta_Blockers_Atenolol	2263	56.12%	83.16%	66.42%	98.67%
ACEi_Lisinopril+Beta_Blockers_Metoprolol+age_60_or_above	2150	80.65%	88.70%	17.21%	96.98%
ARB_Losartan+Thiazide_Hydrochlorothiazide+age_60_or_above	2107	18.27%	67.30%	23.02%	89.18%
ACEi_Enalapril+age_60_or_above	1989	0.00%	8.95%	21.32%	85.27%
CAD_Risk+age_60_or_above	1899	18.27%	0.00%	0.00%	91.21%
age_60_or_above+ckd	1885	40.95%	0.00%	0.00%	94.85%
CCB_Amlodipine+black	1835	4.52%	67.79%	5.83%	95.75%
ACEi_Enalapril	1809	54.39%	22.22%	77.67%	95.63%

Supplementary Figure S11: Number of HTN decision points, grouped by filter variable cohorts, and the percentage that have personalized treatment options with statistically significantly better outcome (green), with fewer medications (purple), with the same number medications (yellow), and with more medications (red).

Filter Variable Cohort	numTotal	Significantly Better Treatment Option	Treatment Option with Fewer Medications	Treatment Option with Same Medication Count	Treatment Option with More Medications
Biguanides_Metformin	33685	97.19%	97.62%	95.71%	100.00%
All Guideline Variables False	17417	100.00%	0.00%	0.00%	100.00%
Biguanides_Metformin+Sulfonylureas_Glipizide	14701	87.84%	100.00%	76.26%	98.93%
Biguanides_Metformin+Sulfonylureas_glimepiride	9378	11.61%	81.50%	83.12%	98.84%
Biguanides_Metformin+Insulin_Insulin_Glargine	9211	59.93%	81.27%	12.00%	97.06%
Biguanides_Metformin+Sulfonylureas_Glyburide	7296	48.23%	96.41%	75.38%	96.15%
Biguanides_Metformin+Insulin_Insulin_Glargine+Insulin_Insulin_Lispro	5010	0.84%	74.45%	0.00%	71.38%
Insulin_Insulin_Glargine	4990	18.68%	91.22%	48.66%	87.19%
Insulin_Insulin_Glargine+Insulin_Insulin_Lispro	3947	2.03%	85.63%	0.00%	64.10%
Biguanides_Metformin+Insulin_Insulin_Glargine+Sulfonylureas_Glipizide	3487	41.81%	96.24%	24.49%	73.30%
BMI_below_25+Biguanides_Metformin	3479	22.59%	74.07%	0.00%	98.39%
Sulfonylureas_Glipizide	3435	76.74%	87.83%	81.95%	95.31%
Sulfonylureas_glimepiride	2052	5.56%	72.76%	46.00%	76.95%
Biguanides_Metformin+Insulin_Insulin_Glargine+Sulfonylureas_Glyburide	1833	4.09%	76.16%	46.54%	43.10%
BMI_below_25	1823	54.69%	0.00%	0.00%	93.80%
Biguanides_Metformin+DPP_4_inhibitors_sitagliptin+Sulfonylureas_Glipizide	1810	0.00%	51.66%	7.90%	38.67%
Sulfonylureas_Glyburide	1690	81.30%	88.34%	36.39%	96.92%
BMI_below_25+Biguanides_Metformin+Sulfonylureas_Glipizide	1613	12.09%	75.08%	0.00%	11.16%
Biguanides_Metformin+Insulin_Insulin_Lispro	1593	2.95%	58.32%	14.88%	11.80%
Biguanides_Metformin+Insulin_Insulin_Glargine+Sulfonylureas_glimepiride	1550	0.00%	85.61%	0.00%	32.39%
Insulin_Insulin_Lispro	1516	2.90%	19.20%	0.00%	86.41%
Biguanides_Metformin+DPP_4_inhibitors_sitagliptin	1223	27.72%	57.32%	40.15%	93.87%
BMI_below_25+Biguanides_Metformin+Sulfonylureas_glimepiride	1025	7.51%	90.34%	0.00%	9.95%
Insulin_Insulin_Glargine+Sulfonylureas_Glipizide	979	62.82%	83.25%	0.00%	84.37%
Biguanides_Metformin+Sulfonylureas_Glipizide+TZDs_pioglitazone	940	0.00%	28.94%	34.89%	43.72%
Biguanides_Metformin+Sulfonylureas_Glyburide+TZDs_pioglitazone	930	0.00%	25.48%	6.99%	70.54%
BMI_below_25+Biguanides_Metformin+Sulfonylureas_Glyburide	902	4.32%	84.15%	6.54%	44.24%
BMI_below_25+Biguanides_Metformin+Insulin_Insulin_Glargine	864	0.00%	31.94%	0.00%	8.33%
Biguanides_Metformin+DPP_4_inhibitors_sitagliptin+Sulfonylureas_glimepiride	842	16.75%	80.52%	0.00%	7.01%
Biguanides_Metformin+TZDs_pioglitazone	674	57.57%	84.27%	0.00%	93.62%
Biguanides_Metformin+Insulin_Insulin_Glargine+Insulin_Insulin_Lispro+Sulfonylureas_Glipizide	651	0.00%	19.66%	0.00%	0.00%
BMI_below_25+Insulin_Insulin_Glargine	575	1.04%	29.39%	0.00%	45.57%
Biguanides_Metformin+Insulin_insulin_detemir	547	0.00%	61.24%	0.00%	19.20%
Insulin_Insulin_Glargine+Sulfonylureas_glimepiride	539	0.00%	7.24%	0.00%	0.00%
Biguanides_Metformin+GLP_1_agonists_exenatide	525	1.52%	1.90%	38.48%	79.81%
Biguanides_Metformin+DPP_4_inhibitors_sitagliptin+Sulfonylureas_Glyburide	513	0.78%	55.36%	28.07%	46.20%
eGFR_below_30	512	4.30%	0.00%	0.00%	75.00%
Insulin_Insulin_Glargine+eGFR_below_30	502	0.00%	10.56%	0.00%	9.56%

Supplementary Figure S12: Number of T2DM decision points, grouped by filter variable cohorts, and the percentage that have personalized treatment options with statistically significantly better outcome (green), with fewer medications (purple), with the same number medications (yellow), and with more medications (red).

Filter Variable Cohort	numTotal	Significantly Better Treatment Option	Treatment Option with Fewer Medications	Treatment Option with Same Medication Count	Treatment Option with More Medications
All Guideline Variables False	345740	100.00%	0.00%	0.00%	100.00%
statin_simvastatin	52058	97.03%	0.00%	100.00%	99.00%
T2D	27090	100.00%	0.00%	0.00%	100.00%
statin_atorvastatin	23781	0.00%	0.00%	15.34%	50.64%
statin_pravastatin	23590	97.47%	0.00%	98.52%	97.44%
ASCVD	11038	100.00%	0.00%	0.00%	100.00%
T2D+statin_simvastatin	10955	23.13%	0.53%	100.00%	99.47%
T2D+statin_atorvastatin	10230	6.22%	0.00%	80.80%	82.26%
Fibric_Acid_Derivative	5300	96.87%	5.40%	94.36%	97.23%
statin_rosuvastatin	5150	0.00%	0.00%	74.80%	78.97%
T2D+statin_pravastatin	5036	81.45%	0.00%	98.57%	92.97%
ASCVD+statin_atorvastatin	4113	0.00%	2.43%	23.51%	31.68%
ASCVD+statin_simvastatin	3131	6.52%	0.64%	97.25%	75.31%
ASCVD+T2D	2994	88.31%	0.00%	0.00%	95.99%
T2D+statin_rosuvastatin	2840	57.82%	2.61%	95.81%	89.15%
statin_lovastatin	2593	77.55%	0.00%	92.98%	88.55%
ASCVD+statin_pravastatin	2182	47.80%	1.19%	85.33%	78.83%
ASCVD+T2D+statin_atorvastatin	2104	0.00%	8.41%	8.51%	25.52%
Bile_acid_Sequestrants	1647	54.46%	3.16%	47.06%	73.71%
Fibric_Acid_Derivative+T2D	1496	75.74%	77.01%	84.96%	89.37%
ASCVD+statin_rosuvastatin	1445	0.14%	8.58%	71.00%	20.90%
Cholesterol_Absorption_Inhibitor	1061	59.38%	1.13%	74.46%	84.45%
ASCVD+T2D+statin_rosuvastatin	1048	0.76%	36.16%	25.57%	32.92%
ASCVD+T2D+statin_simvastatin	961	0.00%	0.00%	83.35%	48.07%
Fibric_Acid_Derivative+statin_simvastatin	781	24.46%	93.85%	34.06%	0.00%
ASCVD+T2D+statin_pravastatin	750	0.00%	17.60%	37.07%	15.87%
statin_atorvastatin+statin_simvastatin	710	16.20%	85.92%	0.00%	0.00%
Bile_acid_Sequestrants+T2D	604	0.00%	22.02%	13.74%	0.00%
ASCVD+Fibric_Acid_Derivative	581	21.00%	2.41%	0.00%	33.73%
T2D+statin_lovastatin	504	3.77%	0.00%	65.08%	0.00%

Supplementary Figure S13: Number of HL decision points, grouped by filter variable cohorts, and the percentage that have personalized treatment options with statistically significantly better outcome (green), with fewer medications (purple), with the same number medications (yellow), and with more medications (red).
